# Efficient production of gene-edited onion (*Allium cepa*) plants using biolistic delivery of cas9 RNPs and transient expression constructs

**DOI:** 10.1007/s00299-025-03626-3

**Published:** 2025-10-19

**Authors:** Cameron L. De La Mora, Michael J. Havey, Patrick J. Krysan

**Affiliations:** Department of Plant and Agroecosystem Sciences, University of Wisconsin-Madison, Madison, WI USA

**Keywords:** Onion, Gene editing, Ribonucleoproteins, Transient expression, DMR6

## Abstract

**Key message:**

Delivery of Cas9/sgRNA RNPs and DNA vectors designed to transiently express morphogenic regulatory genes or an antibiotic resistance gene enabled efficient recovery of gene edited onion lines.

**Abstract:**

We developed a protocol for efficiently producing gene edited onion plants that does not depend on stable transformation. The process makes use of transient gene expression to enrich for gene editing among plants regenerated from immature embryos bombarded with ribonucleoprotein (RNP) complexes composed of CRISPR-associated protein 9 (Cas9) and single guide RNAs (sgRNAs). We used the Allium cepa Downy Mildew Resistant 6 (AcDMR6) gene as the target gene for our studies and produced a total of 47 onion plants with edited AcDMR6 alleles, including 13 homozygous plants, 12 biallelic plants, 7 heterozygous plants, and 15 chimeric plants. The most effective strategy for producing gene edited onion plants involved co-delivering plasmids encoding a hygromycin-resistance protein and plant developmental regulator genes along with the Cas9/sgRNA RNPs followed by transient Hygromycin selection for 48 h. Using this approach, up to 12% of the regenerated onion plants carried edited alleles of AcDMR6. By comparison, no editing was observed among the 146 plants regenerated from explants transfected with the Cas9/sgRNA RNPs alone. The strategy we describe here for using transient gene expression to enrich for gene editing in onion could potentially be extended to other crop species as well.

**Supplementary Information:**

The online version contains supplementary material available at 10.1007/s00299-025-03626-3.

## Introduction

We report the development of a process for efficiently producing gene edited onion plants that does not involve stable genetic transformation. One of the benefits of using gene editing strategies that are not based on stable genetic transformation is that edited lines can be produced that do not carry unwanted genetic information that needs to be segregated away from the desired gene edits in subsequent generations. One approach to producing gene edited plants without stable genetic transformation is to use ribonucleoprotein complexes (RNPs) composed of CRISPR-associated protein 9 (Cas9) and single guide RNAs (sgRNAs) (Kim et al. 2014; Lin et al. 2014). Delivery of RNPs into plants for gene editing has been described using protoplast-based transfection (Andersson et al. 2018; Badhan et al. 2021; Jiang et al. 2021; Najafi et al. 2023) and biolistic-based transfection (Banakar et al. 2019; Liang et al. 2017; Poddar et al. 2023; Svitashev et al. 2016). To produce gene edited plants using RNPs, the transfected cells need to regenerate into plants. In the case of onion, protocols for efficiently regenerating plants from protoplasts have not been reported. For this reason, we chose to focus on the use of biolistic transfection to introduce RNPs into onion tissue.

A recent study reported the production of gene edited wheat plants via the biolistic transfection of Cas9/sgRNA RNPs into tissue explants followed by regeneration of plants using tissue culture-based methods (Poddar et al. 2023). In that study edited plants were recovered at rates up to 37% of the regenerated plants. A similar approach to producing gene editing plants has also been reported using larch (Ma et al. 2024). In these studies, no selection was used to enrich for gene editing events in the population of regenerated plants produced using tissue culture.

Although directly screening for edited plants in the population of plants regenerated from tissue explants exposed to Cas9/sgRNA RNPs via biolistics has been shown to produce edited lines at a useful frequency for some species as described above, it may be valuable to develop strategies that can enrich for the presence of edited plants in those experiments to make this approach more broadly useful. Because we were interested in developing editing protocols that did not depend on stable genetic transformation, we wanted to explore the possibility of using transient gene expression to enrich for editing in experiments, where Cas9/sgRNA RNPs are delivered to tissue explants using biolistic transfection. We specifically explored two different approaches to using transient gene expression to enrich for editing: (1) transient expression of developmental regulator genes and (2) transient antibiotic selection.

Our rationale for exploring the transient expression of developmental regulator genes as a strategy to enrich for gene editing is based on many examples in the literature, where the ectopic expression of plant developmental regulator genes has been shown to improve plant transformation. This list of genes includes *BABY BOOM* (*BBM*) (Lowe et al. 2016), *WUSCHEL* (*WUS*) (Hoerster et al. 2020; Lowe et al. 2016), *DNA binding with One Finger 3.4* (*DOF3.4*) and *DOF3.6* (Liu et al. 2023), *LAX PANICLE1* (*LAX1*) (Yu et al. 2023), *WOUND-INDUCED DEDIFFERENTIATION1* (*WIND1*) (Jiang et al. 2024), Wuschel-like homeobox protein 2a (*WOX2a*) (McFarland et al. 2023), *WOX5* (Wang et al. 2022) and *GRF–GIF*, a fusion between *Growth-Regulating Factor* (*GRF*) and *GRF-Interacting Factor* (*GIF*) (Debernardi et al. 2020; Vandeputte et al. 2024). Ectopic expression of these genes in somatic cells of several monocot and dicot species has been shown to enhance one or more of the following processes: cell proliferation, callus formation, regeneration, somatic embryogenesis, and transformation efficiency. Nearly all the studies in which these developmental regulators were evaluated involved stably integrating the expression construct into cells, but we were interested in determining if a transient burst of expression would be sufficient to allow us to enrich for gene editing. Our hypothesis for how this might function is as follows. When a tissue explant is put through tissue culture to achieve plant regeneration, only a small number of the cells in that tissue explant move down the developmental pathway that leads to plant regeneration. Within this context, it is possible that a transient burst of expression of a developmental regulator might jump start the transfected cells in the explant down the path towards regeneration preferentially over non-transfected cells in the same explant (Youngstrom et al. 2025). If Cas9/sgRNA RNPs were co-delivered into the explant along with the developmental regulator, then one might expect gene editing to be enriched in those cells of the explant that are the founder cells for any regenerated plants that form.

Another strategy by which transient gene expression could be used to enrich for gene editing involves transient antibiotic selection, as was recently reported using poplar (Hoengenaert et al. 2023). In that study, biolistic delivery was used to introduce plasmids expressing Cas9, sgRNAs, and a Kanamycin resistance gene into callus tissue from poplar. The calli were then subjected to Kanamycin selection for 2 days and subsequently grown under selection-free tissue culture conditions for the remainder of the experiment. This strategy allowed for the recovery of gene edited poplar plants. Amongst the total regenerated plant population, 3% of the plants were edited and also lacked the Cas9 or Kanamycin resistance plasmids integrated in their genomes. This result demonstrated that transient expression of an antibiotic resistance gene can allow for the enrichment of gene editing in plants regenerated from tissue explants transfected using particle bombardment.

In this study, we provide evidence that transient gene expression can be used to enrich for gene editing in onion by co-delivering Cas9 RNPs along with developmental regulator genes, an antibiotic resistance gene, or a combination of both strategies. No edited lines were identified amongst plants regenerated in our trials that lacked transient expression-based enrichment factors. However, edited lines were recovered from every treatment group that received developmental regulator genes and/or the hygromycin-resistance construct. Furthermore, a large majority of the edited lines we produced showed no evidence of stable DNA integration of functional copies of the developmental regulator genes or hygromycin-resistance genes, suggesting that transient expression of the enrichment factors was sufficient to enhance the frequency of gene editing in the population of regenerated plants.

## Materials and methods

### Plant materials

har-Syn’ is a synthetic onion population produced by intercrossing among plants with chartreuse bulbs (Havey 2020). Plants of Char–Syn were grown in a field on the Kincaid family farm (Palmyra, WI, USA) under standard production conditions. Bulbs were harvested in the fall and vernalized at 4–7 °C for 5 months. Starting on March 1st, and every 10 days thereafter, vernalized bulbs were planted in a greenhouse on the University of Wisconsin (UW) campus in 400 CU plastic pots containing a soilless mix (PROMIX HP Mycorrhizae; Premier Tech Horticulture, USA) with supplemental lighting at 14 h and a temperature of 25 °C during days and 20 °C during nights. Umbels were caged in groups, when possible, as soon as the first flower of an inflorescence shed pollen, then two teaspoons of fly (Musca domestica) pupae (Rincon-Vitova, Ventura CA) were added, and the cage was corked. Fly pupae were added every 3–4 days to have a continuous presence of pollinators up to day 17. Cages were removed on day 23, and several buds were harvested and their seeds removed for examination. Optimal seeds were blackened, but not yet hardened, and could be squeezed to release an immature embryo 2.5–3.5 mm in length. If most of the seeds were at this optimal stage, then the umbel was cut from the plant, rinsed with cool water, and transported to the lab in water to keep the umbels hydrated.

### Transient editing assay using protoplasts

Four sgRNAs were designed using the ChopChop tool (Labun et al. 2019) to target different locations in exons 1 and 2 of the *AcDMR6* gene with the goal of introducing frameshift mutations in the coding sequence. sgRNAs were commercially synthesized (Integrated DNA Technologies, USA) to target the following sequences: sgRNA-1 5’-GGAATTGGAGAGATTACCTG-3’, sgRNA-2 5’-TGAGTGACGAATTGATATCG3’, sgRNA-3 5’-GGTCGATGACTGGAACGTTT3’, and sgRNA-4 5’-GTGGATAGCAATGAAGCCTC-3’. Each sgRNA was tested for its editing efficiency using a transient protoplast editing assay. Briefly, leaf tissue was harvested from 3-week-old onion plants grown in sterile 32 oz culture vessels. Onion protoplasts were isolated using a protocol originally developed for carrots (Meyer et al. 2022). Cas9/sgRNA RNPs were assembled and transfected as described (Yarra and Krysan 2025). Briefly, Cas9 and the *AcDMR6-*sgRNA1 were allowed to assemble by mixing 2 µl of 1 × PBS (phosphate buffered saline) buffer (Corning, Cat#46–013-CM), 2 µl of sgRNA (100 µM), and 2 µl of Cas9–GFP protein (10 µg/µl) (Integrated DNA Technologies, USA 10008161) and incubating the mixture for 10 min at 25 °C. The pre-complexed Cas9/RNPs were delivered to protoplasts via polyethylene glycol mediated transfection as described (Yarra and Krysan 2025). After transection, samples were incubated in the dark for 3 days at 25 °C, and then DNA was extracted from the protoplasts. First, each 1.2 ml of protoplast solution was transferred to a 1.5 ml safe-lock microcentrifuge tube (Eppendorf, Germany 0030123611). Then, the protoplasts were pelleted via centrifugation at 400 g for 5 min. The supernatant was removed by pipetting, and 240 µl of Buffer E (20 mM Tris 8, 2.5 mM EDTA, 25 mM NaCl, 0.05% SDS) was added to each tube. The protoplasts were lysed using 3.2 mm steel beads (Next Advance, USA SSB32) and a Bullet Blender Tissue Homogenizer (Next Advance, USA BBY24MR). After lysing, the tubes were centrifuged at 16,000 g for 2 min. Next, 15 µl of the crude cell extract was added to 360 µl of Milli-Q water. This diluted extract was vortexed and then used as DNA template for PCR using the forward primer 5’-ACGCTTGTCCTTCCCTGTATC-3’ and reverse primer 5’-ACGGTCGGTGCATGGAAT-3’ to amplify a 721 bp region of the *AcDMR6* gene. Sanger sequencing was performed on the amplicons using the forward primer described above, and the Sanger sequence data were used for TIDE analysis to quantify the frequency of indel mutations generated by each sgRNA (Brinkman et al. 2014). Wild-type onion seedling DNA was used to produce control sequence data for TIDE analysis.

### Transient editing assay of tissue transfected using biolistics

Seeds of ‘Char-Syn’ were washed in 70% ethanol for 30 s, rinsed with Milli-Q water, then washed with a 30% v/v bleach (Clorox, USA, 30,966) solution (15 ml of 8.25% bleach + 35 ml Milli-Q water) and plated on ½ strength MS medium. After 3 days, root radicles 3–4 mm were excised and subject to biolistic transfection as described below. Briefly, RNPs were assembled following the same steps described in the previous section, but 1 × PBS was replaced with 3 × NEBuffer r3.1 (NewEnglandBiolabs, USA B6003S). A 10 mg/ml working suspension of 0.6 µm gold microcarriers (Bio-Rad, USA 1652262) was created by suspending 10 mg of gold microcarriers in 1 ml of 100% ethanol. To begin, the 10 mg/ml suspension of gold microcarriers was sonicated for 30 s using a Branson M1800 Mechanical Ultrasonic Cleaner (Branson, USA). 100 µl of 10 mg/ml suspension of gold microcarriers was then pipetted into two sterile 1.5 ml microcentrifuge tubes. The tubes were centrifuged for 1 min at 2,000 g, and the supernatants discarded. The gold microcarriers were then resuspended in 150 µl of sterile, nuclease-free water (Integrated DNA Technologies, USA, 11–05-01–14). The tubes were then sonicated for 10 s, centrifuged, and the supernatants discarded. The gold microcarriers were then resuspended in 40 µl of sterile nuclease-free water. Then 74 µl of 1 × NEBuffer r3.1 was added to each RNP assembly reaction as well as 1 µl of plasmid pStayGold (500 ng/µl) (Fig. S1 and Supplemental File 1), which expresses the StayGold fluorescent protein reporter (Hirano et al. 2022). Each RNP/DNA solution was then combined with 40 µl of the gold microcarrier suspension and mixed gently by pipetting. Next, 1 µl of Transit-2020 (Mirus Bio, USA) was added to each tube and the solutions mixed again by pipetting. The tubes were then placed in an ice bath for 20 min. Next, the tubes were centrifuged for 1 min at 2,000 g and the supernatant was discarded. 40 µl of nuclease-free water was pipetted into the tubes, and they were sonicated for less than half a second. This final suspension of 40 µl was mixed by pipetting and used to load four macrocarriers (Bio-Rad, USA 1652335) with 10 µl each. The 10 µl was dispensed in multiple small drops on the each macrocarrier to facilitate drying. Macrocarriers loaded with droplets of gold suspension were dried in a laminar flow hood at room temperature for 90 min. Biolistic delivery of the sgRNA/Cas9 RNPs and plasmids was performed with a PDS-1000 | He™ system (Bio-Rad, USA). A plate containing 50 excised onion radiclesQ was placed on the platform 6 cm from the stopping screen. A vacuum of 28 inHg was drawn and each plate was bombarded 4 times, twice with 650 PSI rupture discs and twice with a 1100 PSI discs in that order. One-day post-bombardment the samples were transferred to recovery medium plates and screened for pStayGold expression via epifluorescence microscopy. The 4 radicles with the most abundant pStayGold expression were isolated and trimmed at each end to maximize the fraction of StayGold-positive cells in each explant. Radicles 1 and 2 were incubated at 26 °C for 24 h in the dark, and radicles 3 and 4 were incubated at 30 °C for 24 h in the dark. A Bullet Blender Tissue Homogenizer (Next Advance, USA BBY24MR) and a DNeasy Plant Mini kit (Qiagen, Germany, 69,106) were used to extract DNA from each sample. PCR was then performed to amplify a 316 bp region of *AcDMR6* using the forward primer 5’-TCTGGGGAGTTCTTTAAGCTATC-3’ and reverse primer 5’-TGCATGGAATCGTGGCATG-3’. The PCR product was purified using a Qiagen PCR Purification Kit (Qiagen, Germany 28,104) and subjected to amplicon sequencing using Illumina technology by GeneWiz (115 Corporate Blvd, South Plainfield, NJ 07080) using their Amplicon EZ sequencing service. The resulting data were analyzed to determine the number of reads containing indels within a 25 bp window including the expected editing site for each sample.

### Immature embryo extractions

The composition of the macronutrient, micronutrient, and vitamin stock solutions used to make the media in this study are shown in Tables S1 and S2. Immature seeds were extracted from onion umbels ca. 23–25 days after caging under a dissecting microscope and collected in a jar with a damp Kimtech wipe at the bottom. Seeds were sterilized using the same 30% v/v solution of germicidal bleach described above for 10 min on a shaker at 100 rpm, then rinsed 5 times with sterile water. Immature embryos were extracted from seeds in a sterile laminar flow hood under a dissecting microscope. Immature embryos were immediately plated on a piece of sterile Whatman No. 4 filter paper (MilliporeSigma, USA WHA1004125) placed on recovery medium (Table S3) and incubated overnight at 26 °C in the dark. The immature embryos were arranged closely together at the center of the plate to minimize empty space. The next morning, the immature embryos were transferred to osmotic treatment medium (Table S4) by lifting the filter paper to transfer them all simultaneously. Samples were subjected to biolistic transfection 4 h after transfer to the osmotic treatment media.

### Selection-free regeneration of edited lines from bombarded immature embryos

Immature embryos were bombarded using the same parameters as described above for root radicles in the previous section with minor modifications. In this case, each plate contained 50–75 immature embryos. In experiments involving multiple plasmids, constructs were added in equal amounts by pipetting 1 µl of each 500 ng/µl plasmid solution when preparing the gold microcarriers. After bombardment, all samples were incubated on osmotic treatment medium for 16 h overnight at 26 °C in the dark. Immature embryos were then transferred to recovery medium in 5 × 5 arrays, imaged for StayGold expression, and incubated in the dark at 26 °C for 2 weeks to induce callus. Next, samples were transferred to P5 medium (Table S5) in 5 × 5 arrays and incubated for another 2 weeks. After 4 total weeks of callus induction, the samples were transferred to 100 mm × 25 mm Petri dishes (VWR International, USA, 89,107–632) containing SM4 medium (Table S6) to induce shoot regeneration. Large pieces of callus were broken into smaller pieces approximately 10–15 mm in diameter to promote shoot induction. Samples on SM4 medium were subcultured to fresh SM4 medium every 2 weeks for 10–12 weeks. Once shoots were approximately > 20 mm in length, they were transferred to hormone-free ½ strength MS medium (Table S7) to encourage rooting, typically taking 2–4 weeks for roots to establish. Plants were genotyped at this point, and any candidate edited lines were transferred to 32 oz culture vessels (PhytoTech Labs, USA, C221) containing ½ strength MS medium. The tissue culture protocol employed in this study is based on previous studies (Eady et al. 2003, 2008, 1998). The P5 and SM4 media we describe here are updated versions of the originally published recipes (Eady & Davis, personal communication). The recovery medium we describe uses P5 medium recipe as a base, supplemented with 0.7 g/L proline, 1.5 mg/L 2,4-D, and 0.5 g/L MES hydrate. The osmotic treatment medium uses the recovery medium as a base supplemented with 0.2 M D-sorbitol and 0.2 M D-mannitol.

### Transient expression of developmental regulator genes in tissue explants

Constructs designed to express developmental regulator genes were synthesized commercially (Twist Bioscience, USA and Genewiz, USA). The developmental regulator expression plasmids were pBBM, pWUS2, and pGRF–GIF. The pBBM construct expresses a maize optimized CDS of *BABY BOOM2 (ZmBBM2)* (Lowe et al. 2016)*,* pWUS2 expresses a maize optimized CDS of *WUSHCEL2 (ZmWUS2)* (Lowe et al. 2016), and pGRF–GIF expresses the chimeric protein composed of *GROWTH-REGULATING-FACTOR4 (TaGRF4)* and its cofactor *GRF-INTERACTING-FACTOR1 (TaGRF1)* using the *TaGRF4* CDS and *TaGIF1* CDS from wheat (Debernardi et al. 2020)*.* Expression of each developmental regulator gene is driven by the *Cestrum* Yellow Leaf Curling Virus (CmYLCV) promoter (Sahoo et al. 2016) and terminated by the *Arabidopsis* Heat Shock Protein (HSP) terminator (Nagaya et al. 2010). The same promoter and terminator used for these developmental regulator plasmids was also used for pStayGold, the reporter construct we used to evaluate transfection efficiency. Full DNA sequences of these plasmids are provided in Supplemental Files S2, S3, and S4. Plasmid DNA for these constructs are available through Addgene (addgene.org, Watertown, MA) with the following catalog numbers: pStayGold, (Addgene plasmid 243,674), pBBM, (Addgene plasmid 243,675), pWUS2 (Addgene plasmid 243,676), pGRF–GIF (Addgene plasmid 243,677), pHYG (Addgene plasmid 243,678).

### Transient hygromycin selection

The hygromycin-resistance construct used in this study, pHyg, was commercially synthesized (Genewiz, USA) to express the coding sequence of *HYGROMYCIN PHOSPHOTRANSFERASE* (*HPT*) (Waldron et al. 1985). This construct contains the same promoter and terminator described in the previous section for the developmental regulator expression plasmids. The DNA sequence of this plasmid is provided in Supplemental File S5. Immature embryos bombarded with pHYG were transferred 16 h after bombardment from osmotic treatment medium to Hygromycin-B selection medium (Table S8). Immature embryos were imaged for StayGold expression immediately after transfer to the Hygromycin-B plates and were then incubated in the dark at 26 °C for 48 h. After this 48-h selection period, the samples were transferred to recovery medium without Hygromycin-B and the standard tissue culture protocol described above was followed without any additional exposure to Hygromycin-B.

For the embryogenic callus bombardments, immature embryos were plated on recovery medium and allowed to incubate at 26 °C in the dark for 9 days, then subcultured on fresh recovery media plates for an additional 9 days. On day 18, the most robust pieces of callus were selected and positioned for bombardment in the center of an osmotic treatment plate and bombarded as described above following a 4-h incubation on the osmotic treatment plate. 16 h post-bombardment, the samples were transferred from osmotic treatment medium to recovery medium supplemented with 200 mg/L Hygromycin-B, imaged for GFP expression, and incubated at 26 °C in the dark for 48 h. After the selection period, samples were transferred to P5 medium for 2 weeks. At the end of this period different regions of the tissue were distinguishable as dead (white) or healthy (yellow) callus. The dead tissue was excised and discarded so that only healthy, yellow callus was plated moving forward. After 2 more weeks on P5, the small healthy pieces of callus grew into larger pieces of callus 10–30 mm in diameter. Beyond this point, the calli generated from this approach underwent the standard shoot regeneration and root induction protocol described above.

### Restriction enzyme-based assay for gene editing

Indel mutations directed by *AcDMR6*–sgRNA1 are likely to disrupt an *Eco8*1I restriction site present in the 721 bp *AcDMR6* PCR amplicon produced using the PCR primers described above for the protoplast-based transient editing assay. To screen for editing, 8 µl of PCR product was directly mixed with FastDigest 10 × Green Buffer (Thermo Fisher Scientific, USA) and FastDigest *Eco*81I enzyme (Thermo Fisher Scientific, USA) in a final reaction volume of 20 µl and incubated at 37 °C degrees for 30 min. Digestion reactions were then analyzed using agarose gel electrophoresis with 2% (w/v) agarose gels. The presence of *Eco8*1I-resistant PCR products provided evidence of an edited *AcDMR6* allele in the sample.

### Determining genotype of edited onion plants

The workflow to validate and characterize candidate edited lines was as follows. First, candidate edited lines were divided into two preliminary categories based on the *Eco*81I restriction digest results: 1) samples that produced PCR products that were fully resistant to *Eco*81I digestion were classified as homozygous or biallelic mutants. 2) Samples that produced PCR products that were partially resistant to *Eco*81I digestion, characterized by the presence of fragments 721 bp, 534 bp, and 187 bp in size, were classified as heterozygous or chimeric. PCR products from category 1 samples were subjected to Sanger sequencing, and the results were analyzed using DECODR (Bloh et al. 2021). DECODR identified most of these samples as clearly homozygous or biallelic for *AcDMR6* mutations. When DECODR could not resolve the genotype of the sample, samples were analyzed by amplicon sequencing using Oxford Nanopore technology by PlasmidSaurus (2 Tower Pl suite 950, South San Francisco, CA 94080) using their Premium PCR sequencing service. The allele frequency for each variant of *AcMDR6* present in a sample was determined by inspection of the sequencing report produced by PlasmidSaurus. All category 2 samples were analyzed by amplicon sequencing, since restriction digestion and DECODR could not reliably distinguish heterozygous from chimeric genotypes. Samples were characterized as heterozygous if the sequencing results indicated one wild type and one edited allele of AcDMR6 was present at a ratio of 50% edited: 50% WT, ± 8%. Samples with three or more distinct alleles or irregular allele frequencies were classified as chimeric.

### Screening for evidence of stable integration of expression constructs in the genomes of edited plants

To assess whether any functional copies of the developmental regulator genes or the hygromycin-resistance gene had stably integrated into the genomes of regenerated, edited plants, a PCR-based assay was developed targeting the coding regions of the four genes delivered via plasmid bombardment: a 189 bp amplicon of *ZmBBM2* with the forward primer 5’-CCAACTCCGTCGTCTACAAC-3’ and reverse primer 5’-TCTCGTACCCCATCTGAGCA-3’, a 116 bp amplicon of *ZmWUS2* with the forward primer 5’-CCACTTCTTCCGTTGCGATC-3’ and reverse primer 5’-TGTTGCCGATACGTCTTGGT-3’, a 175 bp amplicon of *TaGRF4–TaGIF1* with the forward primer 5’-TGGGACATTCAGTTCAGCTC-3’ and reverse primer 5’-GGTGTTCATGGCCTCAGTCA-3’, and a 177 bp amplicon *HPT* with the forward primer 5’-CTGACCTCTCCCAAACGTCC-3’ and reverse primer 5’-AGAGCATAAGTTCGTCGAGAG-3’. Primers were designed using Primer3 (Untergasser et al. 2012) to amplify regions of each gene’s coding sequence. All primer sets were validated through qPCR using diluted plasmid DNA as a positive control template. DNA from edited lines was used as template for qPCR and products were subjected to a melt curve analysis and gel electrophoresis to determine if they amplified the expected product.

### Propagation of edited onion lines in soil

1.5 ml of Dyna-Gro 7–9-5 plant fertilizer (Dyna-Gro, USA) was mixed with one liter of water and added to four liters of dry, Promix HP Biofungicide + Mycorrhizae soilless mix and mixed by hand for 2 min until the moisture was equally distributed. The soil was aliquoted to pots (6.35 cm L × 6.35 cm W × 8.9 cm H) that fit in a 1020 plastic greenhouse tray (28 cm W × 53 cm L) Large pieces of ½ strength MS media were removed by hand from the roots, and DI water was used to rinse the residual media from the roots before transferring them to soil. Each tray of transplanted onion plants was covered with a sealed humidity dome ca. 30 cm tall. No additional water was added to the trays, while the plants were in the humidity dome. Plants were placed under LED lights at an intensity of 80–90 µmol/m^2^/s at 14 h light/10 h dark at room temperature (25 °C). The vents of the humidity dome were opened after 4 days of being sealed. After 3 days of the vents being open, the lid of the humidity dome was propped open by placing a pencil, where the dome met the tray to allow more air flow for 2–3 days. The humidity dome was completely removed approximately 10 days after the initial transfer of the plants to the soilless mix. For regeneration events that developed into clusters of plants in tissue culture, the clusters were separated into individual plants prior to transfer to soil by gently teasing them apart after their roots were rinsed with DI water. Regenerated plants with approximately 6 leaves were transplanted to the greenhouse for bulb production and eventual flowering.

## Results

### Identification of an efficient sgRNA for editing the onion DMR6 gene

The overall objective of this study was to establish a protocol for efficiently producing gene edited onion plants using ribonucleoprotein (RNP) complexes composed of Cas9 and sgRNAs. To perform this work, we needed to first validate an efficient sgRNA that was able to efficiently target mutations in the onion genome. We chose the onion *AcDMR6* gene as our target gene for two reasons. First, null mutations in this gene have been shown to provide broad spectrum disease resistance in several plant species (Giacomelli et al. 2023; Parajuli et al. 2022; Paula De Toledo Thomazella et al. 2016; Pirrello et al. 2022; Thomazella et al. 2021; Tripathi et al. 2021; Zeilmaker et al. 2015), making *AcDMR6* a scientifically interesting target. Second, mutations in onion *AcDMR6* are not expected to be lethal (Van Damme et al. 2023), so we expected to recover homozygous null mutants if they were produced in our experiments.

**Fig. 1.**
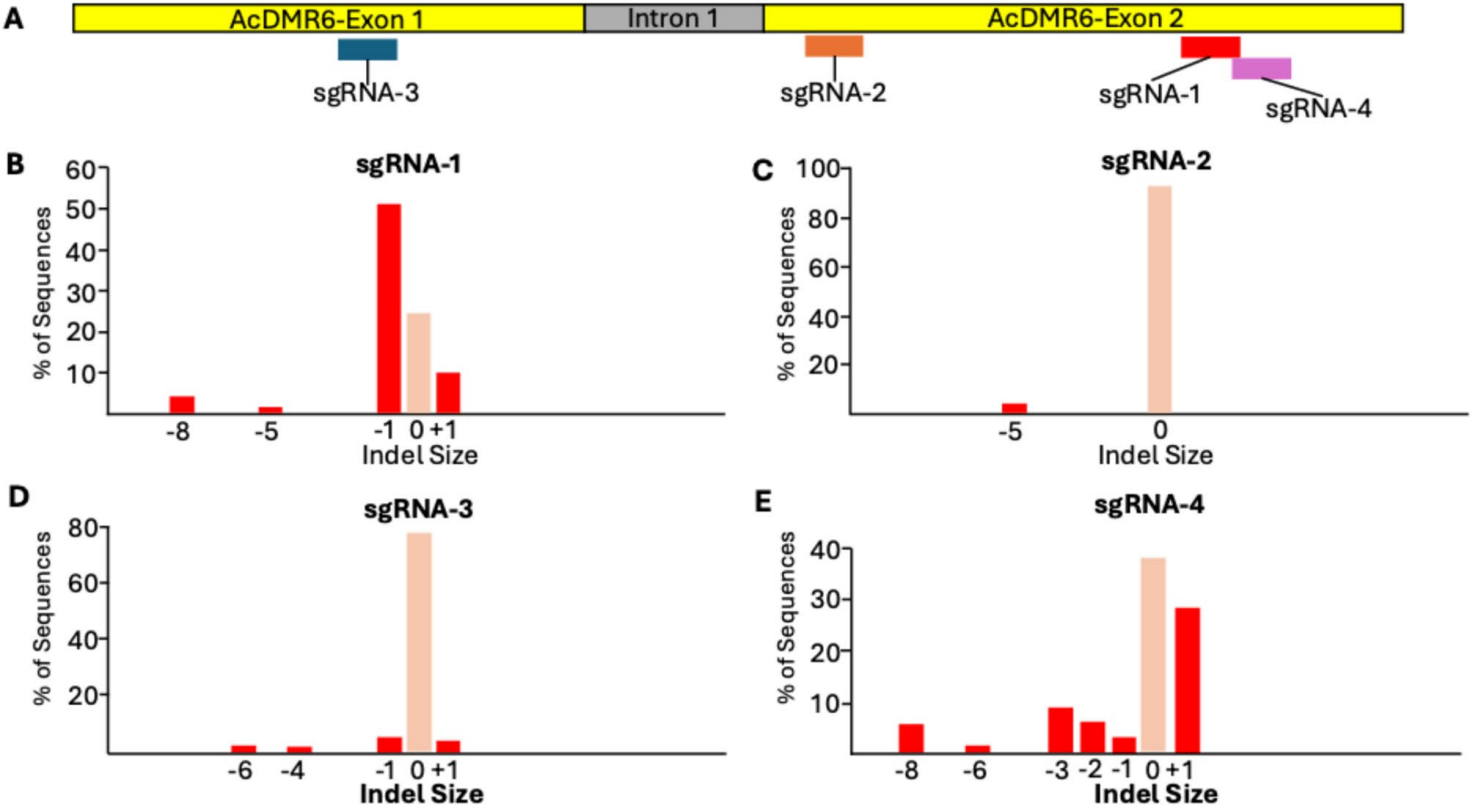
Analysis of sgRNAs used in this study. **A** Locations of the sgRNA target sites within the *AcDMR6* gene. **B–E** Results of onion protoplast transient editing assay. Protoplasts were transfected with Cas9 RNPs produced using the indicated sgRNA, and DNA was extracted from the population of protoplasts 3 days after transfection. PCR targeting the editing site was performed and TIDE analysis of Sanger sequencing traces was used to evaluate gene editing. *X*-axis indicates Indel size and *Y*-axis indicates abundance of each variant as determined by TIDE analysis of PCR amplicons (Brinkman et al. 2014).

Four sgRNAs that target regions within exon 1 or exon 2 of the onion *AcDMR6* gene were synthesized and Cas9/sgRNA RNPs for each were tested for editing efficiency using a protoplast-based transient editing assay (Fig. 1). This analysis indicated that sgRNA-1 produced the highest rate of gene editing among the four sgRNAs tested, with 68% of the copies of the *AcDMR6* gene recovered from the protoplasts sample showing evidence of indel mutations at the expected target site (Fig. 1). Based on this analysis, we chose to use sgRNA-1 for all of the subsequent experiments.

### Using particle bombardment to deliver Cas9/sgRNA RNPs into onion tissue for gene editing

**Fig. 2:**
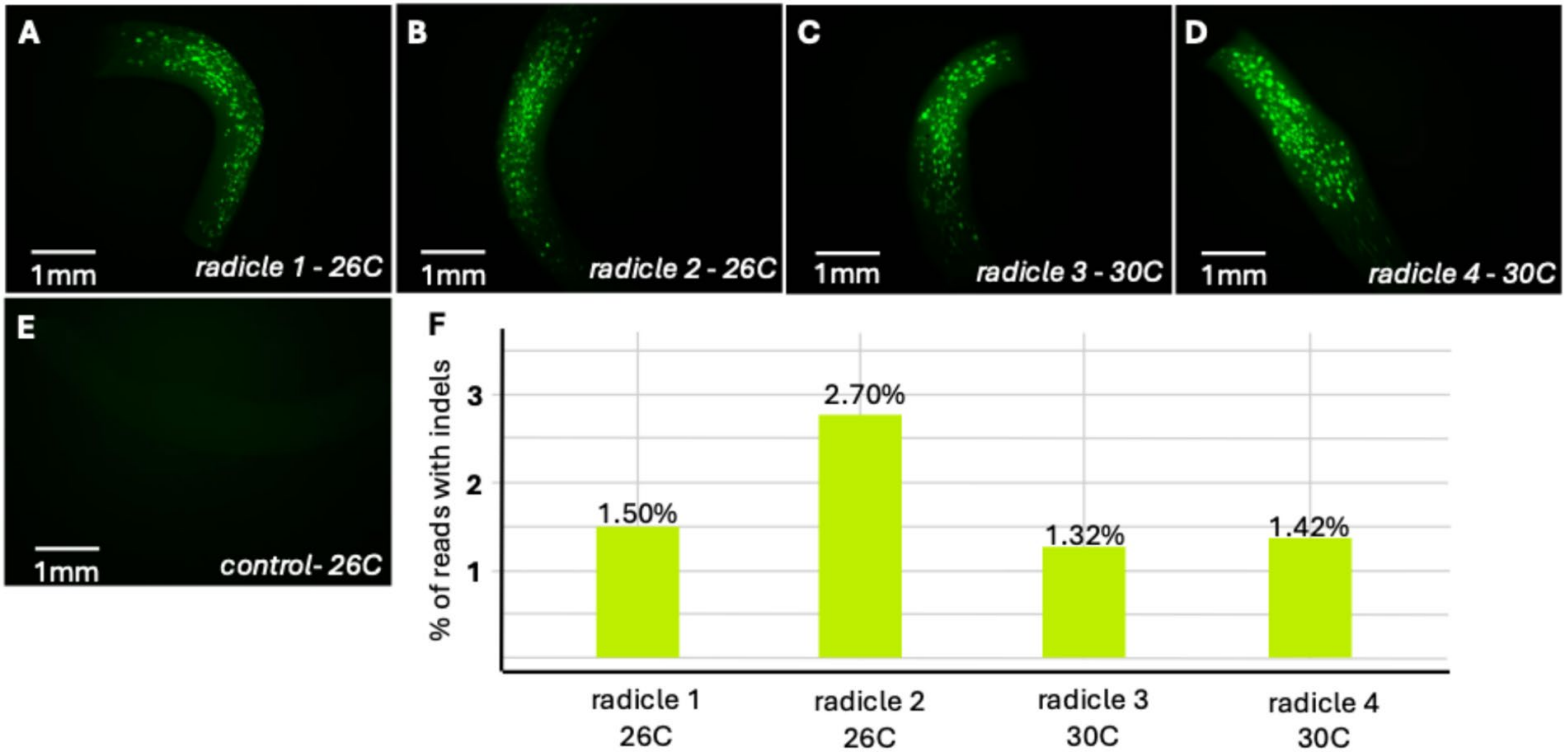
Transient editing assay using onion radicles. Radicles were bombarded with Cas9/AcDMR6 sgRNA-1 RNPs and pStayGold. **A–D** StayGold expression visualized using epifluorescence microscopy 16 h post-bombardment. **E** Control radicle that was not bombarded with pStayGold observed using epifluorescence microscopy. **F** Abundance of Indel mutations in *AcDMR6* for each radicle shown in **A–E**. Incubation temperature post-bombardment is indicated for each sample. Percent of reads with Indels was determined using amplicon sequencing

To determine if Cas9/sgRNA RNPs delivered into onion tissue via biolistic transfection can direct efficient gene editing, we performed a transient gene editing experiment using newly emerged radicles as the tissue explant. For this experiment, we produced Cas9/sgRNA RNPs using the *AcDMR6* sgRNA-1 described above. These RNPs were co-delivered into the radicle explants along with the pStayGold fluorescent protein reporter construct (Fig. S1). Fluorescent protein imaging was performed 1-day post-bombardment to identify four radicles with similar levels of StayGold expression (Fig. 2). Following bombardment, two of these radicles were incubated for 2 days at 26 °C and two of them were incubated for 2 days at 30 °C. Indel mutations within the targeted region of *AcDMR6* occurred at rates ranging from 1.32% to 2.70% in these samples (Fig. 2). Samples incubated at 30 °C did not have higher rates of editing in this experiment, contrary to expectations based on published reports in other plant species (Jiang et al. 2021; Poddar et al. 2023). This experiment demonstrated that Cas9/sgRNA RNPs delivered via biolistic transfection can direct targeted gene editing in onion tissue samples at a detectable rate. Because we did not observe increased editing in the samples incubated at 30 °C in this experiment, we chose to perform the remainder of the experiments at 26 °C.

### Screening for Cas9/sgRNA1 RNP-induced gene editing in regenerated onion plants

We next determined if we could recover gene edited onion plants from tissue explants that had been bombarded with Cas9/sgRNA RNPs targeting the onion *AcDMR6* gene. For these experiments we chose to work with immature embryos as the tissue explant, since this tissue is amenable to plant regeneration (Eady et al. 2003, 2008, 1998; Eady and Lister 1998).

**Fig. 3:**
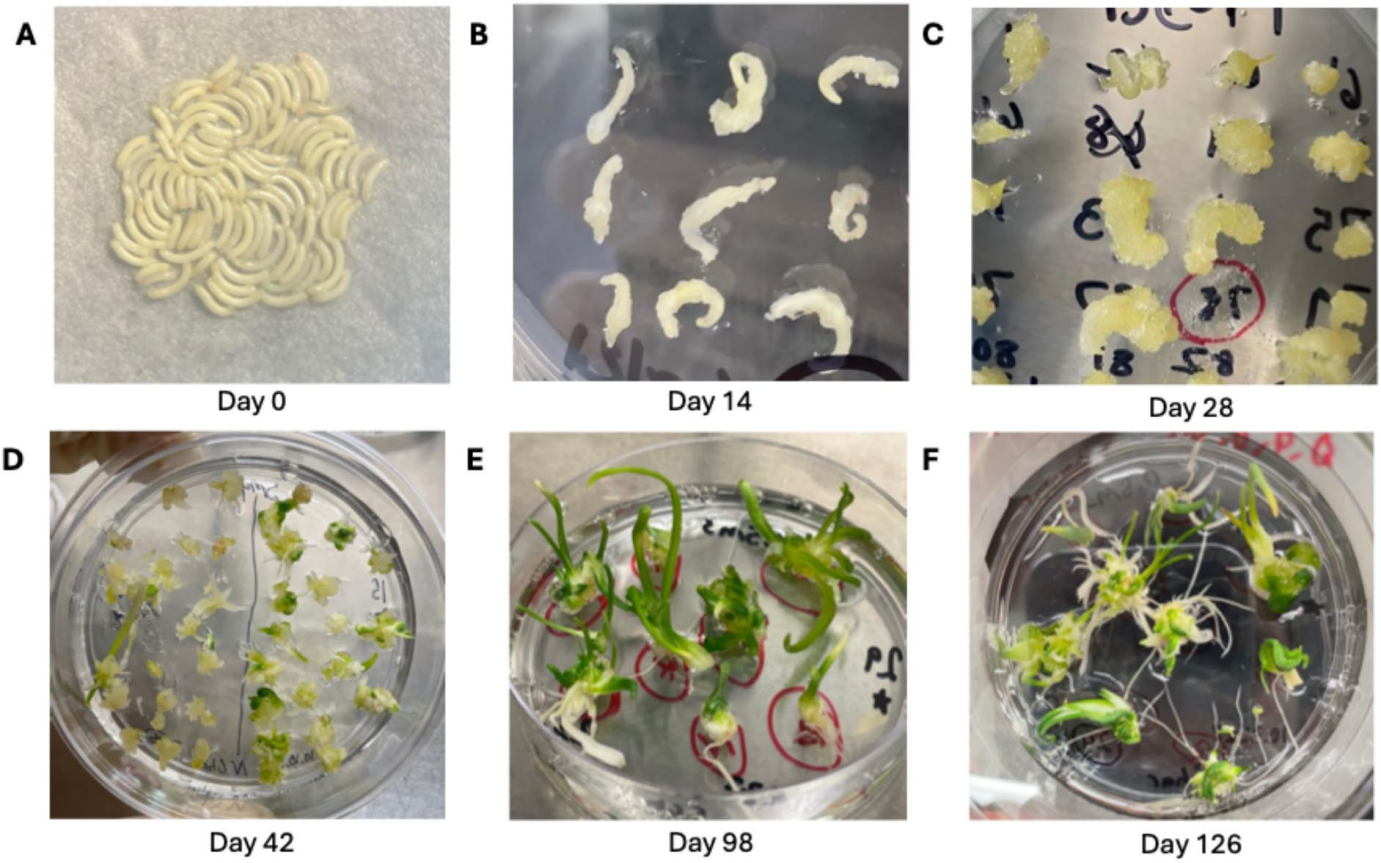
Plant regeneration tissue culture protocol. **A** Freshly isolated immature embryos arranged for bombardment. **B** Calli generated from immature embryos after 2 weeks on recovery medium. **C** Embryogenic calli after 2 weeks on P5 medium. The yellow bumpy properties of these calli indicate that these samples are ready for transfer to SM4 shoot regeneration medium. **D** Calli begin to green and shoots emerge after 2 weeks on SM4 medium. **E** Shoots formed after 10 weeks on SM4 medium. **F** Plants grown on hormone-free ½ strength MS medium for ca. 4 weeks to encourage rooting. Total elapsed time, since immature embryo isolation is indicated under each image

**Fig. 4:**
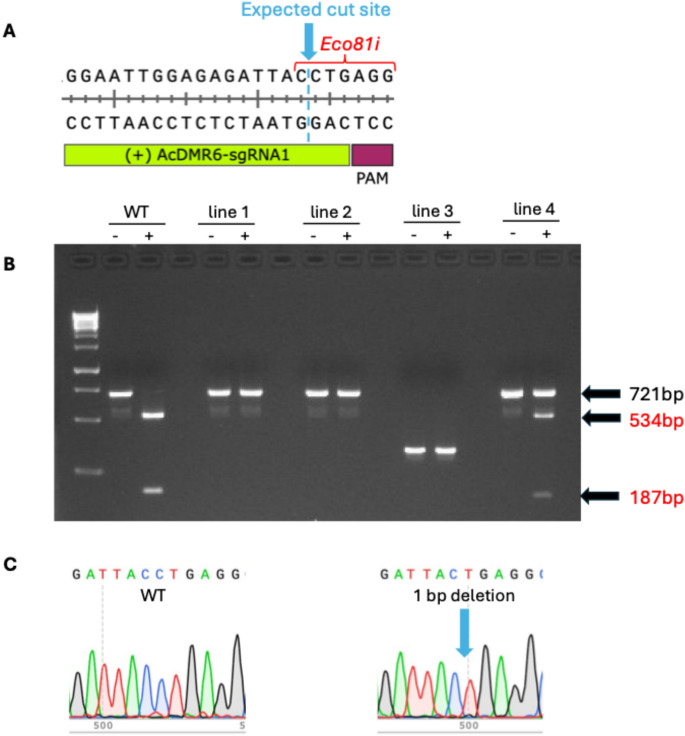
Restriction enzyme assay for *AcDMR6* editing. **A** Position of an *Eco*81I restriction enzyme recognition site with respect to the expected cut site directed by *AcDMR6* sgRNA-1. **B** Agarose gel showing *AcDMR6* PCR products produced from representative edited plants. Reactions were either treated ( +) or not treated (-) with *Eco*81I prior to loading on the gel. “WT” indicates a wild-type control line. Line 1 is homozygous for a 1-bp deletion, Line 2 is biallelic for a 1-bp deletion and a 4-bp deletion, Line 3 is homozygous for a 366 bp deletion, and line 4 was determined to be heterozygous based on follow-up amplicon sequencing. Undigested PCR products are seen at 721 bp, while digestion with *Eco*81I produces fragments of 534 bp and 187 bp. 1 Kb DNA ladder is seen in lane 1 on the gel. **C** Representative Sanger sequencing traces from a wild-type plantlet and a plantlet homozygous for a 1-bp deletion that disrupts the *Eco*81I site

For this experiment, immature embryo explants from onion were co-bombarded with *AcDMR6* sgRNA-1 RNPs along with pStayGold, and the explants were put through a plant regeneration protocol without the use of selectable markers (Fig. 3). Following 3 months of tissue culture, a total of 146 regenerated onion plantlets were produced (Table 1). We use the term “plantlet” to refer to the small, regenerated onion plants that first form in tissue culture. These plantlets were tested for gene editing early in the tissue culture process. In some cases these single plantlets expanded to produce a clump of multiple new plants, but we treated these clumps of plants as a single regeneration event when counting the number of regenerated and edited plantlets produced in our expeirments. To test for the presence of *AcDMR6* gene editing in these plants, a restriction enzyme-based strategy was used (Fig. 4). Briefly, editing of *AcDMR6* using *AcDMR6* sgRNA-1 is expected to disrupt an *Eco*81I restriction site present in the *AcDMR6* amplicon used in our experiments, making PCR products produced from gene edited plants resistant to *Eco*81I digestion. None of the 146 regenerated plantlets we tested produced any *Eco*81I-resistant PCR products, suggesting that gene editing was not present at a detectable level in the regenerated plantlets.

**Table 1:** Edited plants recovered from immature embryo explants

Enrichment plasmids	Explants bombarded	Plantlets regenerated	Regeneration rate	Edited plantlets	% Edited plantlets^a^	Homozygous	Biallelic	Heterozygous	Chimeric
None	261	146	56%	0	0	0	0	0	0
GRF–GIF	126	183	145%	4	2.2%*	2	1	0	1
WUS2	54	60	111%	1	1.7%^NS^	0	0	0	1
BBM + WUS2	108	161	149%	4	2.5%*	1	1	2	0
BBM + GRF–GIF	111	32	29%	1	3.1%*	0	0	0	1
HYG + GRF–GIF	136	157	115%	20	12.7%*	6	4	2	8
HYG + GRF–GIF + BBM	208	31	15%	3	9.7%*	1	0	2	0

^a^“Percent edited plants” was determined by dividing the number of edited plants identified by the total number of plants regenerated for a given treatment type

“*”Indicates that the percentage of edited plants observed is significantly different (two-sample, one-tailed *z* test, *p* < 0.05) than the percentage observed for the explants that were not bombarded with enrichment plasmids

“NS” Indicates that the percentage of edited plants observed is not significantly different (two-sample, one-tailed *z* test, *p* > 0.05) than the percentage of edited plants observed for the explants that were not bombarded with enrichment plasmids

### Expression of developmental regulators to enrich for gene editing in regenerated onion plants

Our failure to recover gene edited onion plants in the experiment described above suggested that edited cells in the bombarded immature embryo explants do not efficiently lead to regenerated plants. We next tested if co-bombardment of plasmids expressing plant developmental regulator genes along with the Cas9/sgRNAs could enrich for gene editing in the regenerated population of plants. The rationale of this experiment is that the transient expression of a developmental regulator could jump start a bombarded cell down the path to regeneration, thereby enriching for editing in the population of cells that enter the regeneration pathway. To test this hypothesis, we constructed expression vectors that placed *BABY BOOM* (*ZmBBM2*) (Lowe et al. 2016), *WUSCHEL* (*ZmWUS2*) (Hoerster et al. 2020; Lowe et al. 2016), or *GRF–GIF*, a fusion between *Growth-Regulating Factor* (*TaGRF4*) and *GRF-Interacting Factor* (*TaGIF1*) (Debernardi et al. 2020; Vandeputte et al. 2024) under the control of the CmYLCV constitutive promoter (Sahoo et al. 2016) (Fig. S1).

For these experiments, *AcDMR6* sgRNA-1 RNPs were co-delivered into immature embryo explants along with pStayGold and one or more of the developmental regulator gene expression plasmids p*BBM*, p*WUS2*, and p*GRF–GIF*. Bombarded explants were then put through the standard tissue culture-based plant regeneration protocol without selection (Fig. 3). The different combinations of developmental regulator plasmids we co-delivered for these experiments were: pGRF–GIF alone, pWUS2 alone, pBBM plus pWUS2, and pBBM plus pGRF–GIF. The regenerated plantlets produced for each trial were tested for evidence of gene editing in *AcDMR6* using the restriction enzyme-based assay described above. Edited plantlets were recovered for all the developmental regulator treatments tested (Table 1). The frequency with which edited plantlets were observed among the populations of regenerated plants ranged from 1.7% for the experiment using pWUS2 alone to a high of 3.1% for the trial testing the combination of pBBM plus pGRF–GIF. For this calculation we divided the number of edited plantlets observed by the total number of regenerated plantlets for a given experiment. Each edited plantlet was considered to be an independent event, because each arose from its own separate piece of callus tissue. Although some of these edited plantlets expanded to produce multiple additional plants during tissue culture, our calculation of editing rate only counted the initial edited plantlets once, not the total number of plants that those events expanded to produce over the course of the tissue culture process. For this experiment, a total of 10 edited plantlets were identified, including 3 homozygous, 2 biallelic, 2 heterozygous, and 3 chimeric mutants. The significance of the observed increase in the frequency of edited plantlets was evaluated using a two-sample, one-tailed *z* test to compare the samples exposed to developmental regulators to the control samples that were not. Using this approach, all of the increases in the rate of recovering edited plantlets were significant (two-sample, one-tailed *z* test, p < 0.05) except for the samples transfected with pWUS2 alone. The allelic state of each mutation in the edited plantlets and whether it appeared to be chimeric was determined using a combination of restriction enzyme testing, Sanger sequencing, and amplicon sequencing as described in the Methods section. It should be noted that the characterization of a plant as having heterozygous versus chimeric mutations should be considered tentative, since this distinction can only be fully resolved by observing the transmission of the edited alleles through sexual generations.

### Using transient hygromycin selection to enrich for gene editing in regenerated onion plants

The results presented above demonstrated that the co-delivery of plasmids expressing developmental regulator genes may enrich for the presence of gene editing in regenerated plants. Although these results were encouraging, the overall rate with which gene edited plantlets were recovered was relatively low. We, therefore, determined if additional enrichment for editing could be achieved using transient antibiotic selection as recently reported for poplar (Hoengenaert et al. 2023). The rationale for this experiment was that transient expression of an antibiotic resistance gene in a transfected cell should provide that cell with transient protection from the antibiotic. If the tissue explant is only exposed to antibiotic selection for 2 days, the transfected cell should be able to survive and then proliferate once selection is removed. By contrast, growth of neighboring cells that were not transfected should be inhibited or killed by the short exposure to high levels of the antibiotic.

For these experiments, *AcDMR6* sgRNA1 RNPs were co-delivered along with pStayGold, pHyg, which confers resistance to Hygromycin, and either pGRF–GIF or pGRF–GIF and pBBM. We included the developmental regulator expression plasmids in these experiments to attempt to build upon the enrichment for gene editing that we observed in the previous experiment. 16 h after bombardment, the tissue explants were transferred to growth media supplemented with 200 mg/L Hygromycin-B and incubated under selection for 48 h. The explants were then transferred to fresh growth media without Hygromycin and subjected to the standard tissue culture process described above without additional antibiotic selection for the remainder of the experiment.

**Fig. 5:**
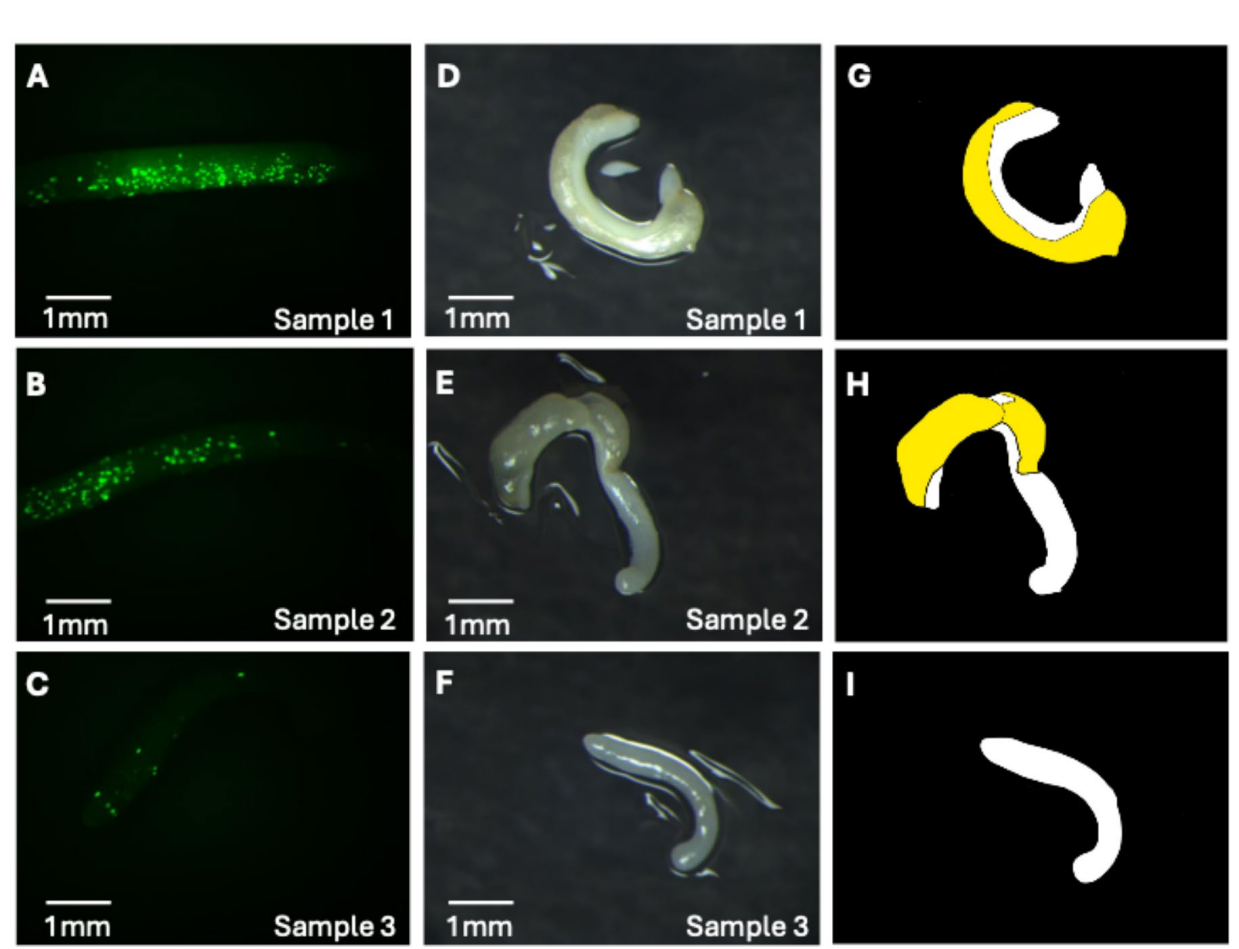
Transient hygromycin selection with immature embryo explants. Immature embryo explants were co-transfected with *AcDMR6*–sgRNA1 RNPs, pHyg, and pGRF–GIF, subjected to 48 h of treatment with Hygromycin-B, and incubated on selection-free media for an additional 24 h. **A–C** StayGold expression visualized 24 h post-transfection. **D–F** Brightfield images of the same explants shown in **A–C** 24 h after Hygromycin treatment was complete. Regions of the explant undergoing cell proliferation appear yellowish, while regions with no cell proliferation appear white. **G–I** Cartoon drawing to highlight the regions of each explant that are undergoing cell proliferation as indicated by yellow coloring. Sample 3 had little StayGold expression and no detectable cell proliferation

Observation of the explants using epifluorescence microscopy 1-day post-bombardment revealed explants with varying degrees of expression of the StayGold fluorescent reporter (Fig. 5). Following 2 days of Hygromycin selection, we observed that cell proliferation correlated with the regions of the transfected explants that displayed StayGold expression. This observation suggested that the transfected cells may be resistant to the Hygromycin treatment when compared with other regions of the same explant. Control explants that were not bombarded showed no evidence of cell proliferation following 48 h Hygromycin treatment. In fact, those control samples did not develop any callus or show any signs of cell proliferation for the full duration of the several-month-long tissue culture process if they were exposed to Hygromycin for 48 h (Fig. S2).

For the samples transfected with RNPs plus the Hygromycin-resistance construct and the developmental regulator plasmids, we recovered a total of 188 regenerated plantlets. *AcDMR6* gene editing was observed in 12.7% of the plantlets regenerated from the explants transfected with the pHyg plus pGRF–GIF (Table 1). For the experiment testing pHyg along with pGRF–GIF and pBBM, 9.7% of the regenerated plantlets were edited. Compared to the control samples that were not transfected with any enrichment plasmids, the rate of edited plantlets in the pHyg plus pGRF–GIF and the pHyg plus pGRF–GIF and pBBM were both significantly higher than that observed for the control that did not produce any edited plantlets (two-sample, one-tailed *z* test, p < 0.05). A total of 23 edited plantlets were produced for these experiments, including 7 homozygous, 4 biallelic, 4 heterozygous, and 8 chimeric.

### Testing transient hygromycin selection using embryogenic callus as the bombarded explant

We observed above that a combination of transient Hygromycin selection plus the expression of a developmental regulator gene can substantially enrich for the presence of gene editing in regenerated plants. In that experiment, immature embryo explants were used for bombardment. Because a previous study that used transient antibiotic selection to enrich for gene editing in plants used embryogenic callus as the explant for bombardment rather than immature embryos (Hoengenaert et al. 2023), we were interested in testing transient Hygromycin selection using onion embryogenic callus as the starting explant.

**Fig. 6:**
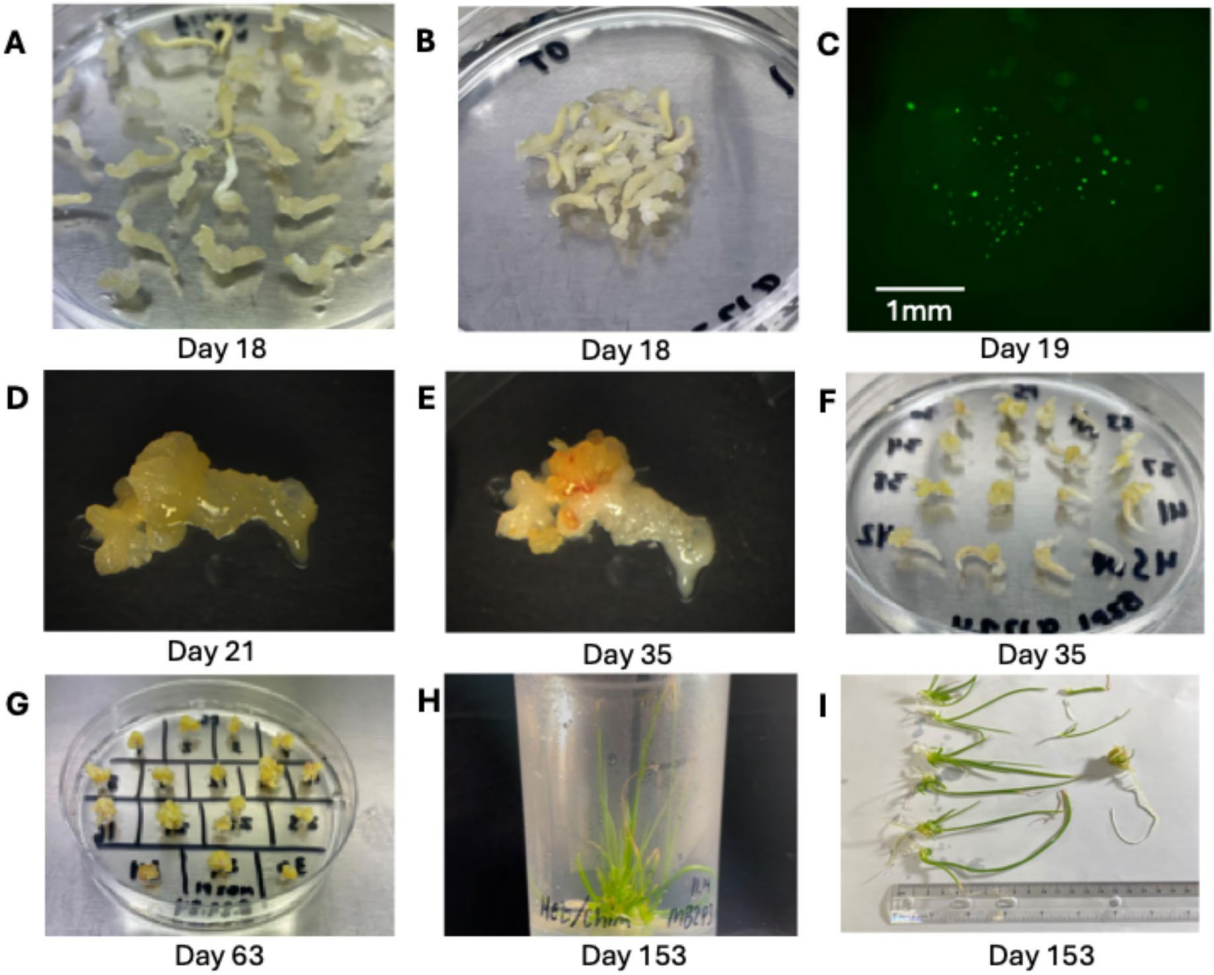
Transient Hygromycin selection with fresh callus. **A** 18-day-old embryogenic callus derived from immature embryo explants. **B** Selected 18-day-old callus arranged for bombardment. **C** StayGold expression in embryogenic callus imaged 16 h after bombardment using epifluorescence microscopy. **D** Bombarded piece of callus following 48 h of Hygromycin selection. **E** Same piece of callus shown in panel D after 2 weeks of incubation on P5 medium. Dead tissue appears white, while healthy tissue appears yellow. **F** Callus after 2 weeks of incubation on P5 medium. **G** Callus after additional growth on P5 medium. **H** Cluster of regenerated plants produced by this system. **I** Individual plantlets that were teased apart from the cluster of plants in panel H. Total elapsed time, since initial immature embryo isolation is indicated under each image

For this experiment we incubated onion immature embryos on callus induction media for 18 days and then selected explants that had well-developed embryogenic callus growth (Fig. 6). *AcDMR6* sgRNA1 RNPs were then co-delivered into this fresh callus along with pStayGold, pHyg, and either pGRF–GIF, pGRF–GIF and pBBM, or no developmental regulator plasmids. Bombarded callus was observed 1-day post-bombardment to determine StayGold expression (Fig. 6) and subjected to a 48-h Hygromycin treatment. Samples were then returned to the standard tissue culture protocol without any additional antibiotic selection.

A total of 232 plantlets were regenerated for these experiments, including 14 plantlets with targeted mutations in *AcDMR6* (Table 2). The frequency with which edited plantlets were recovered in all these trials ranged from 5.8% to 6.4%. The addition of developmental regulator plasmids did not significantly change the rate with which edited plantlets were produced in these experiments when compared to the sample that did not receive any developmental regulator plasmids (two-sample, two-tailed *z* test, *p* < 0.05). The edited plantlets produced in these experiments included 3 homozygous, 6 biallelic, 1 heterozygous, and 4 chimeric.

**Table 2:** Edited plants recovered from callus using transient Hygromycin selection

Enrichment plasmids	Explants bombarded	Plants regenerated	Regeration rate	Edited plantlets	% Edited plantlets^a^	Homozygous	Biallelic	Heterozygous	Chimeric
HYG	79	78	99%	5	6.4%	1	3	0	1
HYG + GRF–GIF	91	50	55%	3	6.0%^NS^	0	2	1	0
HYG + GRF–GIF + BBM	94	104	111%	6	5.8%^NS^	2	1	0	3

^a^ “Percent edited plants” was determined by dividing the number of edited plants identified by the total number of plants regenerated for a given treatment type

“NS” Indicates that the percentage of edited plants observed for the trials involving developmental regulator expression is not significantly different (two-sample, two-tailed *z* test, *p* > 0.05) than the percentage of edited plants observed for the explants that were bombarded with the HYG plasmid but no developmental regulator plasmids

A complete list of all the edited plants produced in this study is presented in Table 3. This table documents the deletion sizes of the edited alleles, as well as the allele ratios observed for plants with more than one allele (Table 3).

**Table 3:** Characteristics of the 47 edited onion plants identified in this study

Sample ID	Enrichment gene	Category	Allele 1	Allele 2	Allele 3	Allele 4	Allele 5	Analysis type
10.9–13	WUS	Chimeric	66% -1 bp	25.85% -3 bp	8.15% WT	/	/	Amplicon Seq^a^
10.2–54	BBM + WUS	Heterozygous	49.18% -3 bp	50.82% WT	/	/	/	Amplicon Seq
10.28–18	BBM + WUS	Biallelic	51.2% -4 bp	48.8% -8 bp	/	/	/	DECODR^b^
10.3–3	BBM + WUS	Homozygous	100%-1 bp	/	/	/	/	Amplicon Seq
9.24–31	BBM + WUS	Heterozygous	47%–3 bp	53% WT	/	/	/	Amplicon Seq
10.1–2	GRF	Chimeric	40.64% WT	32.17% -5 bp	27.2% -5 bp	/	/	Amplicon Seq
10.15–12	GRF	Biallelic	52.4% -5 bp	47.6% -34 bp	/	/	/	DECODR
9.23–16	GRF	Homozygous	100% -108 bp	/	/	/	/	DECODR
9.23–28	GRF	Homozygous	100% -1 bp	/	/	/	/	DECODR
10.18–15	GRF + BBM	Chimeric	76.89% -180 bp	20.65% -58 bp	2.5% -35 bp	/	/	Amplicon Seq
11.1–11	HYG	Chimeric	42.1% -1 bp	16% + 172 bp	41.8% WT	/	/	Amplicon Seq
11.14–27	HYG	Homozygous	100% -1 bp	/	/	/	/	DECODR
11.22–13	HYG	Biallelic	52.68% -6 bp	47.32% -1 bp	/	/	/	Amplicon Seq
11.6–10	HYG	Biallelic	56.5% -1 bp	43.5% -4 bp	/	/	/	DECODR
12.3–14	HYG	Biallelic	58.8% -1 bp	41.2% -4 bp	/	/	/	DECODR
10.14–51	HYG + GRF	Biallelic	50% -5 bp	50% -35 bp	/	/	/	DECODR
10.14–54	HYG + GRF	Homozygous	100% -1 bp	/	/	/	/	Amplicon Seq
10.14–59	HYG + GRF	Chimeric	72.36% WT	17.27% -5 bp	10.37% -1 bp	/	/	Amplicon Seq
10.18–21	HYG + GRF	Homozygous	100% -1 bp	/	/	/	/	DECODR
10.18–27	HYG + GRF	Homozygous	100% -86 bp	/	/	/	/	DECODR
10.18–28	HYG + GRF	Homozygous	100% -1 bp	/	/	/	/	DECODR
10.21–36	HYG + GRF	Biallelic	48.4% -1 bp	51.6% -12 bp	/	/	/	DECODR
10.28–2	HYG + GRF	Chimeric	66% -5 bp	34% -35 bp	/	/	/	DECODR
10.28–3	HYG + GRF	Biallelic	50.4% -1 bp	49.6% -8 bp	/	/	/	DECODR
10.28–7	HYG + GRF	Homozygous	100% -1 bp	/	/	/	/	DECODR
10.7–29	HYG + GRF	Chimeric	16.01% -8 bp	17.5% -1 bp	66.49% WT	/	/	Amplicon Seq
10.7–3	HYG + GRF	Heterozygous	57.5% -1 bp	42.5% WT	/	/	/	Amplicon Seq
10.7–6	HYG + GRF	Chimeric	53.08% -13 bp, + 2 T	36.25% -3 bp	10.67% WT	/	/	Amplicon Seq
10.9–28	HYG + GRF	Chimeric	56.76% WT	25.07% -10 bp	18.17% -3 bp	/	/	Amplicon Seq
11.22–17	HYG + GRF	Heterozygous	57.16% WT	42.84% -1 bp	/	/	/	Amplicon Seq
11.6–13	HYG + GRF	Chimeric	25.1% -5 bp	17.44% -1 bp	57.46% WT	/	/	Amplicon Seq
11.6–14	HYG + GRF	Biallelic	50% -1 bp	50% -8 bp	/	/	/	DECODR
11.7–14	HYG + GRF	Chimeric	45.8% -1 bp	16.72% -3 bp	37.48% -6 bp	/	/	Amplicon Seq
11.8–15	HYG + GRF	Biallelic	59.2% -1 bp	40.8% -10 bp	/	/	/	DECODR
11.8–6	HYG + GRF	Heterozygous	48.43% -1 bp	51.17% WT	/	/	/	Amplicon Seq
12.3–17	HYG + GRF	Biallelic	55.99% -3 bp	44.01% -6 bp	/	/	/	Amplicon Seq
9.25 -5	HYG + GRF	Homozygous	100% -1 bp	/	/	/	/	DECODR
9.30–6	HYG + GRF	Chimeric	66.42% + G	33.58% -1 bp	/	/	/	Amplicon Seq
10.21–21	HYG + GRF + BBM	Chimeric	85.8% -328 bp	14.2% WT	/	/	/	Amplicon Seq
10.31–14	HYG + GRF + BBM	Homozygous	100% -1 bp	/	/	/	/	Plasmidsaurus
10.31–18	HYG + GRF + BBM	Biallelic	51.36% + G	48.64% -6 bp	/	/	/	Amplicon Seq
10.8–24	HYG + GRF + BBM	Heterozygous	57.98% -1 bp	42.02% WT	/	/	/	Amplicon Seq
10.8–34	HYG + GRF + BBM	Heterozygous	51.7% -1 bp	48.3% WT	/	/	/	Amplicon Seq
11.6–16	HYG + GRF + BBM	Homozygous	100% -366 bp	/	/	/	/	DECODR
11.6–18	HYG + GRF + BBM	Chimeric	4.69% -1 bp	3.76% -2 bp	36.2% 6 bp	38.95% -7 bp	16.39% WT	Amplicon Seq
11.6–2	HYG + GRF + BBM	Chimeric	85.5% -3 bp	14.5% + 247 bp	/	/	/	Amplicon Seq
11.8–10	HYG + GRF + BBM	Homozygous	100% -10 bp	/	/	/	/	DECODR

^a^“Amplicon Seq” indicates that amplicon sequencing using Oxford Nanopore technology was used to characterize the genotype of the edited line

^b^“DECODR” indicates that DECODR analysis of Sanger sequencing data was used to characterize the genotype of the edited line

### Screening for the presence of expression constructs in the edited lines

The results described above suggest that the transient expression of a hygromycin-resistance gene and/or developmental regulator genes may enrich for gene editing in regenerated onion plants. If transient expression is sufficient to drive the enrichment for gene editing, then one would expect to recover edited lines that do not have functional copies of the expression plasmids stably inserted in the genome. By contrast, if enrichment depends on long-term expression of the plasmids, then one would expect to find functional copies of the Hygromycin-resistance and/or developmental regulator expression plasmids inserted into the genomes of the edited plants. To explore this question, we designed PCR primers that amplified regions of the coding sequences of all the expression plasmids used in these experiments, including pGRF–GIF, pBBM, pWUS2, and pHyg. PCR reactions were performed using these primers for each of the edited lines produced in our experiments. Evidence of stable integration of DNA from the hygromycin-resistance protein coding sequence in pHyg was observed for 3 out of the 37 edited plants produced using transient Hygromycin selection (Table 4). For the developmental regulator experiments, we found that 4 out of 37 plants had evidence of stable integration of DNA from the *TaGRF4–TaGIF1* protein coding sequence in pGRF–GIF. None of the 13 edited plants recovered using pBBM and none of the 4 plants recovered using pWUS2 showed evidence of stable integration of the complete protein coding sequences for the developmental regulators present in those constructs. The objective of these experiments was to determine if functional copies of the protein coding sequences in each enrichment plasmid had been integrated into the genome of edited plants. The absence of an intact protein coding sequence for a given plasmid does not mean that there are not fragments of that plasmid integrated into the genomes of the edited lines. Determining if the regenerated plants are free of foreign DNA will require additional testing as these experiments were not designed to address that question.

**Table 4:** PCR screen for stable integration of expression vector sequences in edited plants

Sample ID	Explant Bombarded	Mutation type	Enrichment plasmids	Hyg	GRF	BBM	WUS
10.9–13	Immature embryo	Chimeric	WUS	/^a^	/	/	Negative^b^
10.2–54	Immature embryo	Heterozygous	BBM + WUS	/	/	Negative	Negative
10.28–18	Immature embryo	Biallelic	BBM + WUS	/	/	Negative	Negative
10.3–3	Immature embryo	Homozygous	BBM + WUS	/	/	Negative	Negative
9.24–31	Immature embryo	Heterozygous	BBM + WUS	/	/	Negative	Negative
10.1–2	Immature embryo	Chimeric	GRF	/	Negative	/	/
10.15–12	Immature embryo	Biallelic	GRF	/	Negative	/	/
9.23–16	Immature embryo	Homozygous	GRF	/	Negative	/	/
9.23–28	Immature embryo	Homozygous	GRF	/	Negative	/	/
10.18–15	Immature embryo	Chimeric	GRF + BBM	/	Negative	Negative	/
11.1–11	Embryogenic callus	Chimeric	Hyg	Negative	/	/	/
11.14–27	Embryogenic callus	Homozygous	Hyg	Positive^c^	/	/	/
11.22–13	Embryogenic callus	Biallelic	Hyg	Negative	/	/	/
11.6–10	Embryogenic callus	Biallelic	Hyg	Negative	/	/	/
12.3–14	Embryogenic callus	Biallelic	Hyg	Negative	/	/	/
10.14–51	Immature embryo	Biallelic	Hyg + GRF	Negative	Positive	/	/
10.14–54	Immature embryo	Homozygous	Hyg + GRF	Negative	Negative	/	/
10.14–59	Immature embryo	Chimeric	Hyg + GRF	Negative	Positive	/	/
10.18–21	Immature embryo	Homozygous	Hyg + GRF	Negative	Negative	/	/
10.18–27	Immature embryo	Homozygous	Hyg + GRF	Negative	Negative	/	/
10.18–28	Immature embryo	Homozygous	Hyg + GRF	Negative	Negative	/	/
10.21–36	Embryogenic callus	Biallelic	Hyg + GRF	Negative	Negative	/	/
10.28–2	Immature embryo	Chimeric	Hyg + GRF	Negative	Negative	/	/
10.28–3	Immature embryo	Biallelic	Hyg + GRF	Negative	Negative	/	/
10.28–7	Immature embryo	Homozygous	Hyg + GRF	Negative	Negative	/	/
10.7–29	Immature embryo	Chimeric	Hyg + GRF	Negative	Negative	/	/
10.7–3	Immature embryo	Heterozygous	Hyg + GRF	Negative	Negative	/	/
10.7–6	Immature embryo	Chimeric	Hyg + GRF	Negative	Negative	/	/
10.9–28	Immature embryo	Chimeric	Hyg + GRF	Negative	Negative	/	/
11.22–17	Immature embryo	Heterozygous	Hyg + GRF	Negative	Negative	/	/
11.6–13	Immature embryo	Chimeric	Hyg + GRF	Positive	Negative	/	/
11.6–14	Immature embryo	Biallelic	Hyg + GRF	Negative	Negative	/	/
11.7–14	Immature embryo	Chimeric	Hyg + GRF	Negative	Negative	/	/
11.8–15	Immature embryo	Biallelic	Hyg + GRF	Negative	Negative	/	/
11.8–6	Embryogenic callus	Heterozygous	Hyg + GRF	Negative	Negative	/	/
12.3–17	Embryogenic callus	Biallelic	HYG + GRF	Negative	Negative	/	/
9.25–5	Immature embryo	Homozygous	Hyg + GRF	Positive	Positive	/	/
9.30–6	Immature embryo	Chimeric	Hyg + GRF	Negative	Positive	/	/
10.21–21	Embryogenic callus	Chimeric	Hyg + GRF + BBM	Negative	Negative	Negative	/
10.31–14	Embryogenic callus	Homozygous	Hyg + GRF + BBM	Negative	Negative	Negative	/
10.31–18	Embryogenic callus	Biallelic	Hyg + GRF + BBM	Negative	Negative	Negative	/
10.8–24	Immature embryo	Heterozygous	Hyg + GRF + BBM	Negative	Negative	Negative	/
10.8–34	Immature embryo	Heterozygous	Hyg + GRF + BBM	Negative	Negative	Negative	/
11.6–16	Immature embryo	Homozygous	Hyg + GRF + BBM	Negative	Negative	Negative	/
11.6–18	Embryogenic callus	Chimeric	Hyg + GRF + BBM	Negative	Negative	Negative	/
11.6–2	Embryogenic callus	Chimeric	Hyg + GRF + BBM	Negative	Negative	Negative	/
11.8–10	Embryogenic callus	Homozygous	Hyg + GRF + BBM	Negative	Negative	Negative	/

^a^ “/” indicates that the plasmid was not included in that bombardment experiment

^b^ “Negative” indicates that no PCR products were detected

^c^ “Positive” indicates that a PCR product of the expected size was detected

### Propagation of edited onion lines in soil

We identified a total of 47 edited onion plants in tissue culture. After their initial identification as edited plants, we allowed each of these edited plants to grow larger in tissue culture before transferring them to soil. At the point, where these plants were transferred to soil, we observed that 10 of the 47 edited lines initially observed to be a single plant ended up developing into clusters of multiple plants (Fig. 6). These clusters were teased apart into individual plants (Fig. 6), transferred to pots containing soilless mix, and subsequently genotyped. This analysis demonstrated that the plants in each cluster were genotypically identical to the original plant that first emerged in tissue culture, suggesting that they were clonally produced from the same initial editing event. The data shown in Tables 1, 2, and 3 report the frequency of gene editing observed in our initial screen. We later quantified the number of events that led to groups of edited clones being recovered from single initial plants (Table 5).

**Table 5:** Clones identified from plantlets that formed multiple genotypically identical plants

Initial edited plantlet	Initial Genotype	Total number of clones	Putative clone genotype
9.23–16	Homozygous	3	Homozygous
10.15–12	Biallelic	2	Biallelic
11.6–18	Chimeric	2	Chimeric
11.6–16	Homozygous	3	Homozygous
10.8–34	Heterozygous	7	Heterozygous
11.6–2	Chimeric	9	Chimeric
11.8–6	Heterozygous	9	Heterozygous
11.22–6	Heterozygous	9	Heterozygous
12.3–14	Biallelic	3	Biallelic
11.6–10	Biallelic	17	Biallelic

## Discussion

### Enrichment for gene editing using transient expression of developmental regulator genes

Our results indicate that transient expression of developmental regulator genes can increase the frequency of gene edited onion plants using biolistic transfection. No edited plants were produced when Cas9/sgRNA RNPs were delivered into immature embryos without any plasmids designed to provide enrichment for editing, while edited lines were recovered when different combinations of the development regular genes GRF–GIF, BBM, and WUS2 were expressed. The rate with which edited lines occurred among the regenerated plantlets for these experiments ranged from 1.7% to 3.1%. Our working model for how transient expression of a developmental regulator might enhance gene editing in the population of regenerated plants is as follows. When a tissue explant is subjected to tissue culture conditions, a relatively small number of cells in that explant proceed down the developmental pathway that leads to regeneration of a new plant. If a plasmid encoding a developmental regulator gene is transiently expressed in one of those cells that may be sufficient to stimulate that cell towards regeneration preferentially over other cells in the explant. By co-delivering Cas9/sgRNA RNPs into those same cells, it may be possible to preferentially drive edited cells towards regeneration. Further research will be needed to determine which developmental regulators are best suited to enrich for gene editing in this system and what the underlying mechanism is responsible for enrichment.

### Enrichment for gene editing using transient antibiotic expression

While the enrichment for gene editing we observed using developmental regulator constructs was encouraging, we hoped to develop an editing pipeline that produced edited lines at a rate higher than the 3.1% we observed with that strategy. We explored whether transient expression of a hygromycin-resistance gene could allow higher rates of enrichment for editing. These experiments build upon the previously reported success of using transient Kanamycin selection to enrich for editing in poplar (Hoengenaert et al. 2023). In our case, we observed up to 12.7% of the regenerated plants were edited when transient Hygromycin selection was used in conjunction with expression of the TaGRF4–TaGIF1 developmental (Debernardi et al. 2020) regulator using immature embryos as the starting material. Further research will be needed to determine the relative contributions of transient Hygromycin selection and transient TaGRF4–TaGIF1 expression to this enrichment. Regardless, we have demonstrated that enrichment strategies can be used to efficiently produce gene edited onion plants without stable genetic transformation.

One observation was that exposing onion immature embryos to Hygromycin for 48 h is sufficient to block cell proliferation under the tissue culture conditions used in these experiments. The control samples did not show any cell growth or cell division and did not form any callus when they were exposed to Hygromycin for 48 h. By contrast, similar explants put into tissue culture without Hygromycin treatment rapidly produce large amounts of callus. These results suggest that a short 48-h Hygromycin treatment effectively kills cells in an immature embryo explant, thereby providing us with a strong genetic selection for the expression of a resistance gene. Further work will be needed to determine if the resistance phenotype provided by transient expression of the hygromycin-resistance gene acts in a cell-autonomous manner. Regardless, this selection appears to allow for substantial enrichment for gene editing in regenerated plants.

### Transient expression of enrichment factors appears to be sufficient to enhance the recovery of edited plants

The hypothesis underlying our experiments is that transient expression of developmental regulators or an antibiotic resistance gene should allow for the enrichment of gene editing in regenerated plants. With biolistic transfection, an introduced plasmid can have two different fates. It can remain extrachromosomal and drive transient expression of the encoded gene until the plasmid is degraded or lost from the nucleus during cell division. Alternatively, the plasmid can become linearized and stably incorporated into the genome of the cell. In this case expression of the gene encoded by the plasmid could persist in the cell lineages derived from the initial cell in which the plasmid integrated. If transient expression was not sufficient to provide the enrichment for gene editing that we observed in this study, then we would expect that most, if not all, of the edited plants recovered in our experiments should have intact copies of the expression units for the developmental regulators and/or hygromycin-resistance gene stably incorporated into their genomes. To explore this possibility, we used PCR to screen for the presence of the protein coding sequence of each of the enrichment factors used in our experiments. We observed that ca. 10% of the edited lines produced using transient Hygromycin selection contained a copy of the hygromycin-resistance coding sequence. For the developmental regulator constructs, ca. 10% of the lines that used *TaGRF4–TaGIF1* (Debernardi et al. 2020) for enrichment had a portion of that coding sequence, while no copies of the *ZmWUS2* (Lowe et al. 2016) nor *ZmBBM2* (Lowe et al. 2016) regions were found. Taken together, these results suggest that stable incorporation of the plasmids encoding the enrichment factors is not necessary to enrich for gene editing in this system.

In the case of using transient Kanamycin selection to enrich for gene editing in poplar, 57% of the edited plants carried a portion of the Kanamycin resistance gene (Hoengenaert et al. 2023). Because there are several differences between the gene editing pipelines used for poplar and onion it is difficult to determine why the rates of DNA integration are different in these two cases. Further research will be needed to better understand what factors determine the frequency of stable DNA integration when transient antibiotic selection is used to enrich for gene editing.

The PCR-based analysis to screen for functional copies of the transient expression constructs in the edited onion lines was not designed to determine if these edited lines are transgene-free. The purpose of the experiment was to explore if transient gene expression was sufficient to drive enrichment for gene editing. To determine if these lines are transgene-free, one would need to perform more exhaustive analysis using either a battery of PCR primers that span the entirety of the plasmids used in each experiment or whole-genome sequencing to search for the presence of any foreign DNA. That analysis was beyond the scope of the current study.

### Production of homozygous, biallelic, heterozygous, and chimeric lines

Among the 47 edited onion plants recovered in this study, ca. 28% were homozygous for the edited allele, ca. 25% were biallelic, ca. 15% were heterozygous, and ca. 32% appeared to be chimeric. These determinations were made using a combination of restriction enzyme analysis, Sanger sequencing, and amplicon sequencing. From a practical perspective, the fact that over 50% of the recovered plants were homozygous or biallelic means that phenotypic studies can be carried out on the regenerated lines without having to go through an additional generation to allow a heterozygous mutation to segregate homozygous progeny. In addition, because stable genetic transformation is not part of our editing pipeline, it should be possible to identify transgene-free edited lines in the population of regenerated plants.

### Edited lines recovered in tissue culture often produce clones during the culturing process

We observed that initial edited plants identified in tissue culture often developed into clusters of edited clonal propagules. This phenomenon presents an opportunity to immediately produce multiple clones of edited plants. Clones were identified from ca. 22% of the total regeneration events that produced edited plantlets, suggesting this phenomenon occurs at a useful rate. The most extreme instance of recovering edited clones was observed with a group of 17 plants derived from the same initial edited plantlet identified in tissue culture. In this example, the initial plantlet identified in tissue culture was found to be biallelic with a 1-bp deletion on one chromosome and a 4-bp deletion on the other. When it was time to transfer this initial plantlet out of tissue culture and into soilless mix, this plantlet had morphed into a cluster of 17 plants. Each of these 17 plants was transferred into a separate pot, and genotype analysis indicated that they are all clones of the initial biallelic plantlet.

## Conclusion

Further testing will be needed to determine the phenotypic effects of the edited *AcDMR6* alleles produced in this study and to study the genetic transmission of these alleles. Examples from other plant species have indicated that plants homozygous for *DMR6* knockout mutations can display resistance to a range of microbial pathogens (Giacomelli et al. 2023; Parajuli et al. 2022; Paula De Toledo Thomazella et al. 2016; Pirrello et al. 2022; Thomazella et al. 2021; Tripathi et al. 2021; Zeilmaker et al. 2015), so it will be interesting to study the disease susceptibility of these edited onion plants to a panel of pathogens. Although chimeric plants represented only ca. 1/3 of the edited plants produced in our experiments, it will be important to follow the transmission of edited alleles into the next generation with these chimeric plants. The gene editing pipeline that we describe provides an efficient process for producing gene edited onion plants without the need for stable genetic transformation. The strategy of using transient gene expression to enrich for the presence of gene editing in the population of regenerated plants should be readily applicable to other crop species.

## Supplementary Information

Below is the link to the electronic supplementary material.

Supplementary file1 (DOCX 434 kb)

## Data Availability

The data sets generated during the current study are available from the corresponding author on reasonable request.
