# Supplementary material for: Efficient production of gene-edited onion (Allium cepa) plants using biolistic delivery of cas9 RNPs and transient expression constructs
Source: Plant Cell Rep. 2025 Oct 19;44(11):243. doi: 10.1007/s00299-025-03626-3 (PMC12535934; doi:10.1007/s00299-025-03626-3)
Supplement: Supplementary file 1 — Supplementary file1 (DOCX 434 kb) [file 299_2025_3626_MOESM1_ESM.docx]

**Table S1. Recipes for BDS stock solutions**

| Reagent | Molar mass | Amount |
| --- | --- | --- |
| **1 liter of 20x BDS Macronutrients** | | |
| Potassium nitrate. | 101.10 g/mol | 50.6 g |
| Ammonium nitrate. | 80.04 g/mol | 6.4 g |
| Magnesium sulfate heptahydrate | 246.47 g/mol | 4.94 g |
| Ammonium phosphate monobasic | 115.03 g/mol | 4.6 g |
| Sodium phosphate monobasic dihydrate | 156.01 g/mol | 3.44 g |
| Calcium chloride dihydrate | 147.01 g/mol | 3 g |
| Ammonium sulfate | 132.14 g/mol | 2.68 g |
| **1 liter of 200x BDS Micronutrients** | | |
| Sodium EDTA | 336.21 g/mol | 7.45 g |
| Ferrous sulfate heptahydrate | 278.01 g/mol | 5.57 g |
| Manganese sulfate tetrahydrate | 223.06 g/mol | 2.64 g |
| Boric acid | 61.83 g/mol | 0.6 g |
| Zinc sulfate heptahydrate | 287.56 g/mol | 0.4 g |
| Potassium iodide | 166.0 g/mol | 0.15 g |
| Cupric sulfate pentahydrate | 249.69 g/mol | 0.078 g |
| Sodium molybdate dihydrate | 241.95 g/mol | 0.05 g |
| Cobalt chloride hexahydrate | 237.93 g/mol | 0.005 g |
| **1 liter of 200x BDS modified vitamins** | | |
| Myo-inositol | 180.16 g/mol | 10 g |
| Glycine | 75.07 g/mol | 250 mg |
| Thiamine HCl | 337.27 g/mol | 50 mg |
| Pyridoxine HCl | 205.64 g/mol | 50 mg |
| Folic acid | 441.4 g/mol | 50 mg |
| Biotin | 244.31 g/mol | 5 mg |
| Nicotinic acid | 123.11 g/mol | 0.5 mg |

**Table S2. Recipes for MS stock solutions**

| Reagent | Molar Mass | Amount |
| --- | --- | --- |
| **1 liter of 20x MS Macronutrients** | | |
| Potassium nitrate | 101.10 g/mol | 38 g |
| Ammonium nitrate | 80.04 g/mol | 33 g |
| Calcium chloride dihydrate | 147.01 g/mol | 8.8 g |
| Magnesium sulfate heptahydrate | 246.47 g/mol | 7.4 g |
| Monopotassium phosphate | 136.09 g/mol | 3.4 g |
| **1 liter of 200x MS Micronutrients** | | |
| Manganese sulfate tetrahydrate | 223.06 g/mol | 4.46 g |
| Zinc sulfate heptahydrate | 287.56 g/mol | 1.72 g |
| Potassium iodide | 166.0 g/mol | 0.166 g |
| Boric acid | 61.83 g/mol | 1.24 g |
| Sodium molybdate dihydrate | 241.95 g/mol | 0.05 g |
| Cupric sulfate pentahydrate | 249.69 g/mol | 0.005 g |
| Cobalt chloride hexahydrate | 237.93 g/mol | 0.005 g |
| **1 liter of 200x MS modified vitamins** | | |
| Myo-inositol | 180.16 g/mol | 10 g |
| Thiamine HCl | 337.27 g/mol | 50 mg |
| Pyridoxine HCl | 205.64 g/mol | 50 mg |
| Nicotinic acid | 123.11 g/mol | 0.5 mg |
| Glycine | 75.07 g/mol | 250 mg |
| Biotin | 244.31 g/mol | 5 mg |
| Folic acid | 441.4 g/mol | 50 mg |
| **1 liter of 200x MS iron** | | |
| EDTA FeNa salt | 367.05 g/mol | 7.34 g |

**Table S3. Recipe for 1 liter of recovery medium**

| Reagent | Molar Mass | Amount |
| --- | --- | --- |
| 20x BDS macronutrient stock solution | n/a | 50 ml |
| 200x BDS micronutrient stock solution | n/a | 5 ml |
| 200x BDS modified vitamin stock solution | n/a | 5 ml |
| Picloram | 241.46 g/mol | 5 mg |
| 2,4-D | 221.04 g/mol | 1.5 mg |
| Sucrose | 342.3 g/mol | 30 g |
| Proline | 115.13 g/mol | 700 mg |
| MES hydrate | 195.24 g/mol | 500 mg |
| Casein Hydrolysate | CAS # 65072-00-6 | 50 mg |
| pH | n/a | 6 |
| Gelrite | CAS # 71010-52-1 | 4 g |

**Table S4. Recipe for 1 liter of osmotic treatment medium**

| Reagent | Molar Mass | Amount |
| --- | --- | --- |
| 20x BDS macronutrient stock solution | n/a | 50 ml |
| 200x BDS micronutrient stock solution | n/a | 5 ml |
| 200x BDS modified vitamin stock solution | n/a | 5 ml |
| Picloram | 241.46 g/mol | 5 mg |
| 2,4-D | 221.04 g/mol | 1.5 mg |
| Sucrose | 342.3 g/mol | 30 g |
| Proline | 115.13 g/mol | 700 mg |
| MES hydrate | 195.24 g/mol | 500 mg |
| Casein Hydrolysate | CAS # 65072-00-6 | 50 mg |
| D-mannitol | 182.17 g/mol | 36.43 g |
| D-sorbitol | 182.17 g/mol | 36.43 g |
| pH | n/a | 6 |
| Gelrite | CAS # 71010-52-1 | 4 g |

**Table S5. Recipe for 1 liter of P5 medium**

| Reagent | Molar Mass | Amount |
| --- | --- | --- |
| 20x BDS macronutrient stock solution | n/a | 50 ml |
| 200x BDS micronutrient stock solution | n/a | 5 ml |
| 200x BDS modified vitamin stock solution | n/a | 5 ml |
| Picloram | 241.46 g/mol | 5 mg |
| Sucrose | 342.3 g/mol | 30 g |
| Casein Hydrolysate | CAS # 65072-00-6 | 50 mg |
| pH | n/a | 6 |
| Gelrite | CAS # 71010-52-1 | 4 g |

**Table S6. Recipe for 1 liter of SM4 Shoot induction medium**

| Reagent | Molar Mass | Amount |
| --- | --- | --- |
| 20x BDS macronutrient stock solution | n/a | 50 ml |
| 200x MS micronutrient stock solution | n/a | 5 ml |
| 200x MS iron stock solution | n/a | 5 ml |
| 200x Modified BDS vitamin stock solution | n/a | 5 ml |
| Kinetin | 215.21 g/mol | 3 mg |
| Adenine hemisulfate | 184.17 g/mol | 84 mg |
| Sucrose | 342.3 g/mol | 30 g |
| pH | n/a | 5.8 |
| Gelrite | CAS # 71010-52-1 | 4 g |

**Table S7. Recipe for 1 liter of ½ strength MS rooting medium.**

| Reagent | Molar Mass | Amount |
| --- | --- | --- |
| 20x MS macronutrient stock solution | n/a | 25 ml |
| 200x MS micronutrient stock solution | n/a | 2.5 ml |
| 200x MS iron stock solution | n/a | 2.5 ml |
| 200x MS vitamin stock solution | n/a | 5 ml |
| Sucrose | 342.3 g/mol | 30 g |
| pH (adjust with KOH) | n/a | 5.8 |
| Gelrite | CAS # 71010-52-1 | 3.5 g |

**Table S8. Recipe for 1 liter of 200 mg/L Hygromycin-B selection medium**

| Reagent | Molar Mass | Amount |
| --- | --- | --- |
| 20x BDS macronutrient stock solution | n/a | 50 ml |
| 200x BDS micronutrient stock solution | n/a | 5 ml |
| 200x BDS modified vitamin stock solution | n/a | 5 ml |
| Picloram | 241.46 g/mol | 5 mg |
| 2,4-D | 221.04 g/mol | 1.5 mg |
| Sucrose | 342.3 g/mol | 30 g |
| Proline | 115.13 g/mol | 700 mg |
| MES hydrate | 195.24 g/mol | 500 mg |
| Casein Hydrolysate | CAS # 65072-00-6 | 50 mg |
| Hygromycin-B | 527.5 g/mol | 200 mg |
| pH | n/a | 6 |
| Gelrite | CAS # 71010-52-1 | 4 g |

**Fig S1.** Transient expression constructs used in this study. All constructs use a promoter from the cestrum yellow lead curling virus (CmYLCV) and a terminator from an *Arabidopsis thaliana* heat shock protein (HSP). **A)** pStayGold contains the CDS for the StayGold fluorescent protein reporter. **B)** pGRF-GIF contains the CDS for a chimera of *GROWTH REGULATING FACTOR* and *GROWTH GRF INTERACTING FACTOR* (*GRF-GIF*), **C)** pBBM contains the CDS for *BABY BOOM2* (*BBM2*), **D)** pWUS2 contains the CDS for *WUSHCEL2* (*WUS2*), and **E)** pHyg contains the CDS for *HYGROMYCIN PHOSPHOTRANSFERASE* (HPT). The ori and KanR sequences are used to propagate the plasmids in *E. coli*. The total size of each plasmid in base pairs (bp) is indicated.

**Fig S2.** Effect of 48 hour Hygromycin selection on callus formation. **A-C)** Immature embryo explants photographed one month after a 48 hour transient Hygromycin selection. **A)** Control explant that did not undergo Hygromycin treatment. **B)** Control explant that was not transfected with pHyg, but did receive a 48 hour Hygromycin treatment. **C)** Explant that was transfected with pHyg and subjected to a 48 hour Hygromycin selection. Images are representative examples of results typically observed for these experiments.

**Supplemental File 1**

LOCUS pStayGold 3750 bp DNA circular SYN 22-APR-2025

DEFINITION CmY_StayGold in pTwist Kan High Copy.

ACCESSION .

VERSION .

KEYWORDS .

SOURCE synthetic DNA construct

ORGANISM synthetic DNA construct

REFERENCE 1 (bases 1 to 3750)

AUTHORS .

TITLE Direct Submission

JOURNAL Exported Apr 22, 2025 from SnapGene 8.0.3

https://www.snapgene.com

COMMENT This file was created using tools provided by Twist Bioscience

FEATURES Location/Qualifiers

source 1..3750

/mol_type="other DNA"

/organism="synthetic DNA construct"

source join(611..3750,1..610)

/organism="CmY_StayGold in pTwist Kan High Copy"

misc_feature 611..1074

/label=CMY 9.11 Promoter

CDS 1085..1738

/codon_start=1

/label=Stay Gold Brassica Optimized

/translation="MASTPFKFQLKGTINGKSFTVEGEGEGNSHEGSHKGKYVCTSGKL

PMSWAALGTSFGYGMKYYTKYPSGLKNWFHEVMPEGFTYDRHIQYKGDGSIHAKHQHFM

KNGTYHNIVEFTGQDFKENSPVLTGDMNVSLPNEVQHIPRDDGVECPVTLLYPLLSDKS

KCVEAHQNTICKPLHNQPAPDVPYHWIRKQYTQSKDDTEERDHICQSETLEAHL"

misc_feature 1739..2195

/label=At HSP Term

rep_origin complement(2440..3028)

/direction=LEFT

/label=ori

/note="high-copy-number ColE1/pMB1/pBR322/pUC origin of

replication"

CDS complement(join(3164..3750,1..229))

/codon_start=1

/gene="aph(3')-Ia"

/product="aminoglycoside phosphotransferase"

/label=KanR

/note="confers resistance to kanamycin in bacteria or G418

(Geneticin(R)) in eukaryotes"

/translation="MSHIQRETSCSRPRLNSNMDADLYGYKWARDNVGQSGATIYRLYG

KPDAPELFLKHGKGSVANDVTDEMVRLNWLTEFMPLPTIKHFIRTPDDAWLLTTAIPGK

TAFQVLEEYPDSGENIVDALAVFLRRLHSIPVCNCPFNSDRVFRLAQAQSRMNNGLVDA

SDFDDERNGWPVEQVWKEMHKLLPFSPDSVVTHGDFSLDNLIFDEGKLIGCIDVGRVGI

ADRYQDLAILWNCLGEFSPSLQKRLFQKYGIDNPDMNKLQFHLMLDEFF"

ORIGIN

1 ccgtcagcca gtttagtctg accatctcat ctgtaacatc attggcaacg ctacctttgc

61 catgtttcag aaacaactct ggcgcatcgg gcttcccata caatcgatag attgtcgcac

121 ctgattgccc gacattatcg cgagcccatt tatacccata taaatcagca tccatgttgg

181 aatttaatcg cggcctcgag caagacgttt cccgttgaat atggctcata acaccccttg

241 tattactgtt tatgtaagca gacagtttta ttgttcatga tgatatattt ttatcttgtg

301 caatgtaaca tcagagattt tgagacacaa cgtggctttc ccccgccgct ctagaactag

361 tggatccaaa taaaacgaaa ggctcagtcg aaagactggg cctttcgttt tatctgttgt

421 ttgtcgcatt atacgagacg tccaggttgg gatacctgaa acaaaaccca tcgtacggcc

481 aaggaagtct ccaataactg tgatccacca caagcgccag ggttttccca gtcacgacgt

541 tgtaaaacga cggccagtca tgcataatcc gcacgcatct ggaataagga agtgccattc

601 cgcctgacct tggcagacat actgtcccac aaatgaagat ggaatctgta aaagaaaacg

661 cgtgaaataa tgcgtctgac aaaggttagg tcggctgcct ttaatcaata ccaaagtggt

721 ccctaccacg atggaaaaac tgtgcagtcg gtttggcttt ttctgacgaa caaataagat

781 tcgtggccga caggtggggg tccaccatgt gaaggcatct tcagactcca ataatggagc

841 aatgacgtaa gggcttacga aataagtaag ggtagtttgg gaaatgtcca ctcacccgtc

901 agtctataaa tacttagccc ctccctcatt gttaagggag caaaatctca gagagatagt

961 cctagagaga gaaagagagc aagtagccta gaagtagtca aggcggcgaa gtattcaggc

1021 aggtggccag gaagaagaaa agccaagacg acgaaaacag gtaagagcta agctttcctg

1081 caggatggca tcaaccccgt tcaagtttca gttgaagggc actattaacg gtaaatcatt

1141 tacggtggag ggggagggcg aggggaacag tcacgagggt agtcacaaag gcaaatatgt

1201 atgtacttca gggaaacttc cgatgtcttg ggcggcactt ggcaccagct ttggctacgg

1261 catgaaatat tacacgaaat atccctcagg cctgaagaat tggtttcacg aagtcatgcc

1321 cgagggattt acctatgacc gtcacattca atacaagggc gatggatcaa ttcatgcgaa

1381 acatcagcac tttatgaaga atggcactta tcacaacata gttgagttta ctggtcaaga

1441 ttttaaggaa aattcccccg tcttgactgg tgatatgaat gtcagcttgc caaatgaggt

1501 gcaacatatt ccgcgtgacg atggcgtaga gtgtcctgtc acgctccttt acccccttct

1561 tagtgataag agtaaatgcg tcgaggccca ccaaaacacg atctgtaagc cgctgcacaa

1621 tcagcctgcc cccgatgtac cttatcattg gattagaaaa caatatactc aatcgaaaga

1681 tgatacggaa gagagagatc atatttgcca atccgagact ttggaggccc atctttgaat

1741 atgaagatga agatgaaata tttggtgtgt caaataaaaa gcttgtgtgc ttaagtttgt

1801 gtttttttct tggcttgttg tgttatgaat ttgtggcttt ttctaatatt aaatgaatgt

1861 aagatctcat tataatgaat aaacaaatgt ttctataatc cattgtgaat gttttgttgg

1921 atctcttctg cagcatataa ctactgtatg tgctatggta tggactatgg aatatgatta

1981 aagataagat gggctcatag agtaaaacga ggcgagggac ctataaacct cccttcatca

2041 tgctatttca tgatctattt tataaaataa agatgtagaa aaaagtaagc gtaataaccg

2101 caaaacaaat gatttaaaac atggcacata atgaggagat taagttcggt ttacgtttat

2161 tttagtacta attgtaacgt gagactacgt atcggaggct aggtggaggc tcagtgatga

2221 taagtctgcg atggtggatg catgtgtcat ggtcatagct gtttcctgtg tgaaattgtt

2281 atccgctcag agggcacaat cctattccgc gctatccgac aatctccaag acattaggtg

2341 gagttcagtt cggcgtatgg catatgtcgc tggaaagaac atgtgagcaa aaggccagca

2401 aaaggccagg aaccgtaaaa aggccgcgtt gctggcgttt ttccataggc tccgcccccc

2461 tgacgagcat cacaaaaatc gacgctcaag tcagaggtgg cgaaacccga caggactata

2521 aagataccag gcgtttcccc ctggaagctc cctcgtgcgc tctcctgttc cgaccctgcc

2581 gcttaccgga tacctgtccg cctttctccc ttcgggaagc gtggcgcttt ctcatagctc

2641 acgctgtagg tatctcagtt cggtgtaggt cgttcgctcc aagctgggct gtgtgcacga

2701 accccccgtt cagcccgacc gctgcgcctt atccggtaac tatcgtcttg agtccaaccc

2761 ggtaagacac gacttatcgc cactggcagc agccactggt aacaggatta gcagagcgag

2821 gtatgtaggc ggtgctacag agttcttgaa gtggtggcct aactacggct acactagaag

2881 aacagtattt ggtatctgcg ctctgctgaa gccagttacc ttcggaaaaa gagttggtag

2941 ctcttgatcc ggcaaacaaa ccaccgctgg tagcggtggt ttttttgttt gcaagcagca

3001 gattacgcgc agaaaaaaag gatctcaaga agatcctttg atcttttcta cggggtctga

3061 cgctctattc aacaaagccg ccgtcccgtc aagtcagcgt aaatgggtag ggggcttcaa

3121 atcgtccgct ctgccagtgt tacaaccaat taacaaattc tgattagaaa aactcatcga

3181 gcatcaaatg aaactgcaat ttattcatat caggattatc aataccatat ttttgaaaaa

3241 gccgtttctg taatgaagga gaaaactcac cgaggcagtt ccataggatg gcaagatcct

3301 ggtatcggtc tgcgattccg actcgtccaa catcaataca acctattaat ttcccctcgt

3361 caaaaataag gttatcaagt gagaaatcac catgagtgac gactgaatcc ggtgagaatg

3421 gcaaaagctt atgcatttct ttccagactt gttcaacagg ccagccatta cgctcgtcat

3481 caaaatcact cgcatcaacc aaaccgttat tcattcgtga ttgcgcctga gcgagacgaa

3541 atacgcgatc gctgttaaaa ggacaattac aaacaggaat cgaatgcaac cggcgcagga

3601 acactgccag cgcatcaaca atattttcac ctgaatcagg atattcttct aatacctgga

3661 atgctgtttt cccggggatc gcagtggtga gtaaccatgc atcatcagga gtacggataa

3721 aatgcttgat ggtcggaaga ggcataaatt

//

**Supplemental File 2**

LOCUS pBBM 5687 bp DNA circular SYN 14-JAN-2025

DEFINITION synthetic circular DNA

ACCESSION .

VERSION .

KEYWORDS .

SOURCE synthetic DNA construct

ORGANISM synthetic DNA construct

REFERENCE 1 (bases 1 to 5687)

AUTHORS .

TITLE Direct Submission

JOURNAL Exported Apr 22, 2025 from SnapGene 8.0.3

https://www.snapgene.com

FEATURES Location/Qualifiers

source 1..5687

/note="/dnas_title=pCMY-ZmBBM2 in pUC-GW-Kan"

/organism="synthetic DNA construct"

misc_feature 416..879

/label=CMY 9.11 Promoter

CDS 890..3019

/codon_start=1

/label=ZmBBM2 CDS

/translation="MATVNNWLAFSLSPQELPPSQTTDSTLISAATADHVSGDVCFNIP

QDWSMRGSELSALVAEPKLEDFLGGISFSEQHHKSNCNLIPSTSSTVCYASSAASTGYH

HQLYQPTSSALHFADSVMVASSAGVHDGGSMLSAAAANGVAGAASANGGGIGLSMIKNW

LRSQPAPMQPRAAAAEGAQGLSLSMNMAGTTQGAAGMPLLAGERARAPESVSTSAQGGA

VVVTAPKEDSGGSGVAGALVAVSTDTGGSGGASADNTARKTVDTFGQRTSIYRGVTRHR

WTGRYEAHLWDNSCRREGQTRKGRQVYLGGYDKEEKAARAYDLAALKYWGATTTTNFPV

SNYEKELEDMKHMTRQEFVASLRRKSSGFSRGASIYRGVTRHHQHGRWQARIGRVAGNK

DLYLGTFSTQEEAAEAYDIAAIKFRGLNAVTNFDMSRYDVKSILDSSALPIGSAAKRLK

EAEAAASAQHHHAGVVSYDVGRIASQLGDGGALAAAYGAHYHGAAWPTIAFQPGAATTG

LYHPYAQQPMRGGGWCKQEQDHAVIAAAHSLQDLHHLNLGAAGAHDFFSAGQQAAAAAA

MHGLASIDSASLEHSTGSNSVVYNGGVGDSNGASAVGSGGGYMMPMSAAGATTTSAMVS

HEQMHARAYDEAKQAAQMGYESYLVNAENNGGGRMSAWGTVVSAAAAAAASSNDNIAAD

VGHGGAQLFSVWNDT"

misc_feature 3020..3476

/label=At HSP Term

rep_origin complement(3913..4501)

/direction=LEFT

/label=ori

/note="high-copy-number ColE1/pMB1/pBR322/pUC origin of

replication"

CDS complement(4672..5487)

/codon_start=1

/gene="aph(3')-Ia"

/product="aminoglycoside phosphotransferase"

/label=KanR

/note="confers resistance to kanamycin in bacteria or G418

(Geneticin(R)) in eukaryotes"

/translation="MSHIQRETSCSRPRLNSNMDADLYGYKWARDNVGQSGATIYRLYG

KPDAPELFLKHGKGSVANDVTDEMVRLNWLTEFMPLPTIKHFIRTPDDAWLLTTAIPGK

TAFQVLEEYPDSGENIVDALAVFLRRLHSIPVCNCPFNSDRVFRFAQAQSRMNNGLVDA

SDFDDERNGWPVEQVWKEMHKLLPFSPDSVVTHGDFSLDNLIFDEGKLIGCIDVGRVGI

ADRYQDLAILWNCLGEFSPSLQKRLFQKYGIDNPDMNKLQFHLMLDEFF"

ORIGIN

1 tcgcgcgttt cggtgatgac ggtgaaaacc tctgacacat gcagctcccg gagactgtca

61 cagcttgtct gtaagcggat gccgggagca gacaagcccg tcagggcgcg tcagcgggtg

121 ttggcgggtg tcggggctgg cttaactatg cggcatcaga gcagattgta ctgagagtgc

181 accatatgcg gtgtgaaata ccgcacagat gcgtaaggag aaaataccgc atcaggcgcc

241 attcgccatt caggctgcgc aactgttggg aagggcgatc ggtgcgggcc tcttcgctat

301 tacgccagct ggcgaaaggg ggatgtgctg caaggcgatt aagttgggta acgccagggt

361 tttcccagtc acgacgttgt aaaacgacgg ccagtgaatt gacgcgtatt gggattggca

421 gacatactgt cccacaaatg aagatggaat ctgtaaaaga aaacgcgtga aataatgcgt

481 ctgacaaagg ttaggtcggc tgcctttaat caataccaaa gtggtcccta ccacgatgga

541 aaaactgtgc agtcggtttg gctttttctg acgaacaaat aagattcgtg gccgacaggt

601 gggggtccac catgtgaagg catcttcaga ctccaataat ggagcaatga cgtaagggct

661 tacgaaataa gtaagggtag tttgggaaat gtccactcac ccgtcagtct ataaatactt

721 agcccctccc tcattgttaa gggagcaaaa tctcagagag atagtcctag agagagaaag

781 agagcaagta gcctagaagt agtcaaggcg gcgaagtatt caggcaggtg gccaggaaga

841 agaaaagcca agacgacgaa aacaggtaag agctaagctt tcctgcagga tggccactgt

901 gaacaactgg ctcgctttct ccctctcccc gcaggagctg ccgccctccc agacgacgga

961 ctccacgctc atctcggccg ccaccgccga ccatgtctcc ggcgatgtct gcttcaacat

1021 cccccaagat tggagcatga ggggatcaga gctttcggcg ctcgtcgcgg agccgaagct

1081 ggaggacttc ctcggcggca tctccttctc cgagcagcat cacaagtcca actgcaactt

1141 gatacccagc actagcagca cagtttgcta cgcgagctca gctgctagca ccggctacca

1201 tcaccagctg taccagccca ccagctccgc gctccacttc gcggactccg tcatggtggc

1261 ctcctcggcc ggtgtccacg acggcggttc catgctcagc gcggccgccg ctaacggtgt

1321 cgctggcgct gccagtgcca acggcggcgg catcgggctg tccatgatca agaactggct

1381 gcggagccaa ccggcgccca tgcagccgag ggcggcggcg gctgagggcg cgcaggggct

1441 ctctttgtcc atgaacatgg cggggacgac ccaaggcgct gctggcatgc cacttctcgc

1501 tggagagcgc gcacgggcgc ccgagagtgt atcgacgtca gcacagggtg gtgccgtcgt

1561 cgtcacggcg ccgaaggagg atagcggtgg cagcggtgtt gccggtgctc tagtagccgt

1621 gagcacggac acgggtggca gcggcggcgc gtcggctgac aacacggcaa ggaagacggt

1681 ggacacgttc gggcagcgca cgtcgattta ccgtggcgtg acaaggcata gatggactgg

1741 gagatatgag gcacatcttt gggataacag ttgcagaagg gaaggacaaa ctcgtaaggg

1801 tcgtcaagtc tatttaggtg gctatgataa agaggagaaa gctgctaggg cttatgatct

1861 tgctgctctg aagtactggg gtgccacaac aacaacaaat tttccagtga gtaactacga

1921 aaaggagctc gaggacatga agcacatgac aaggcaggag tttgtagcgt ctctgagaag

1981 gaagagcagt ggtttctcca gaggtgcatc catttacagg ggagtgacta ggcatcacca

2041 acatggaaga tggcaagcac ggattggacg agttgcaggg aacaaggatc tttacttggg

2101 caccttcagc acccaggagg aggcagcgga ggcgtacgac atcgcggcga tcaagttccg

2161 cggcctcaac gccgtcacca acttcgacat gagccgctac gacgtgaaga gcatcctgga

2221 cagcagcgcc ctccccatcg gcagcgccgc caagcgtctc aaggaggccg aggccgcagc

2281 gtccgcgcag caccaccacg ccggcgtggt gagctacgac gtcggccgca tcgcctcgca

2341 gctcggcgac ggcggagccc tagcggcggc gtacggcgcg cactaccacg gcgccgcctg

2401 gccgaccatc gcgttccagc cgggcgccgc caccacaggc ctgtaccacc cgtacgcgca

2461 gcagccaatg cgcggcggcg ggtggtgcaa gcaggagcag gaccacgcgg tgatcgcggc

2521 cgcgcacagc ctgcaggacc tccaccactt gaacctgggc gcggccggcg cgcacgactt

2581 tttctcggca gggcagcagg ccgccgccgc agctgcgatg cacggcctgg ctagcatcga

2641 cagtgcgtcg ctcgagcaca gcaccggctc caactccgtc gtctacaacg gcggggtcgg

2701 cgatagcaac ggcgccagcg ccgttggcag cggcggtggc tacatgatgc cgatgagcgc

2761 tgccggagca accactacat cggcaatggt gagccacgag cagatgcatg cacgggccta

2821 cgacgaagcc aagcaggctg ctcagatggg gtacgagagc tacctggtga acgcggagaa

2881 caatggtggc ggaaggatgt ctgcatgggg gaccgtcgtc tctgcagccg cggcggcagc

2941 agcaagcagc aacgacaaca ttgccgccga cgtcggccat ggcggcgcgc agctcttcag

3001 tgtctggaac gacacttgaa tatgaagatg aagatgaaat atttggtgtg tcaaataaaa

3061 agcttgtgtg cttaagtttg tgtttttttc ttggcttgtt gtgttatgaa tttgtggctt

3121 tttctaatat taaatgaatg taagatctca ttataatgaa taaacaaatg tttctataat

3181 ccattgtgaa tgttttgttg gatctcttct gcagcatata actactgtat gtgctatggt

3241 atggactatg gaatatgatt aaagataaga tgggctcata gagtaaaacg aggcgaggga

3301 cctataaacc tcccttcatc atgctatttc atgatctatt ttataaaata aagatgtaga

3361 aaaaagtaag cgtaataacc gcaaaacaaa tgatttaaaa catggcacat aatgaggaga

3421 ttaagttcgg tttacgttta ttttagtact aattgtaacg tgagactacg tatcggatcc

3481 caatggcgcg ccgagcttgg ctcgagcatg gtcatagctg tttcctgtgt gaaattgtta

3541 tccgctcaca attccacaca acatacgagc cggaagcata aagtgtaaag cctggggtgc

3601 ctaatgagtg agctaactca cattaattgc gttgcgctca ctgcccgctt tccagtcggg

3661 aaacctgtcg tgccagctgc attaatgaat cggccaacgc gcggggagag gcggtttgcg

3721 tattgggcgc tgttccgctt cctcgctcac tgactcgctg cgctcggtcg ttcggctgcg

3781 gcgagcggta tcagctcact caaaggcggt aatacggtta tccacagaat caggggataa

3841 cgcaggaaag aacatgtgag caaaaggcca gcaaaaggcc aggaaccgta aaaaggccgc

3901 gttgctggcg tttttccata ggctccgccc ccctgacgag catcacaaaa atcgacgctc

3961 aagtcagagg tggcgaaacc cgacaggact ataaagatac caggcgtttc cccctggaag

4021 ctccctcgtg cgctctcctg ttccgaccct gccgcttacc ggatacctgt ccgcctttct

4081 cccttcggga agcgtggcgc tttctcatag ctcacgctgt aggtatctca gttcggtgta

4141 ggtcgttcgc tccaagctgg gctgtgtgca cgaacccccc gttcagcccg accgctgcgc

4201 cttatccggt aactatcgtc ttgagtccaa cccggtaaga cacgacttat cgccactggc

4261 agcagccact ggtaacagga ttagcagagc gaggtatgta ggcggtgcta cagagttctt

4321 gaagtggtgg cctaactacg gctacactag aagaacagta tttggtatct gcgctctgct

4381 gaagccagtt accttcggaa aaagagttgg tagctcttga tccggcaaac aaaccaccgc

4441 tggtagcggt ggtttttttg tttgcaagca gcagattacg cgcagaaaaa aaggatctca

4501 agaagatcct ttgatctttt ctacggggtc tgacgctcag tggaacgaaa actcacgtta

4561 agggattttg gtcatgagat tatcaaaaag gatcttcacc tagatccttt taaattaaaa

4621 atgaagtttt aaatcaatct aaagtatata tgagtaaact tggtctgaca gttagaaaaa

4681 ctcatcgagc atcaaatgaa actgcaattt attcatatca ggattatcaa taccatattt

4741 ttgaaaaagc cgtttctgta atgaaggaga aaactcaccg aggcagttcc ataggatggc

4801 aagatcctgg tatcggtctg cgattccgac tcgtccaaca tcaatacaac ctattaattt

4861 cccctcgtca aaaataaggt tatcaagtga gaaatcacca tgagtgacga ctgaatccgg

4921 tgagaatggc aaaagtttat gcatttcttt ccagacttgt tcaacaggcc agccattacg

4981 ctcgtcatca aaatcactcg catcaaccaa accgttattc attcgtgatt gcgcctgagc

5041 gaaacgaaat acgcgatcgc tgttaaaagg acaattacaa acaggaatcg aatgcaaccg

5101 gcgcaggaac actgccagcg catcaacaat attttcacct gaatcaggat attcttctaa

5161 tacctggaat gctgttttcc cagggatcgc agtggtgagt aaccatgcat catcaggagt

5221 acggataaaa tgcttgatgg tcggaagagg cataaattcc gtcagccagt ttagtctgac

5281 catctcatct gtaacatcat tggcaacgct acctttgcca tgtttcagaa acaactctgg

5341 cgcatcgggc ttcccataca atcgatagat tgtcgcacct gattgcccga cattatcgcg

5401 agcccattta tacccatata aatcagcatc catgttggaa tttaatcgcg gcctagagca

5461 agacgtttcc cgttgaatat ggctcatact cttccttttt caatattatt gaagcattta

5521 tcagggttat tgtctcatga gcggatacat atttgaatgt atttagaaaa ataaacaaat

5581 aggggttccg cgcacatttc cccgaaaagt gccacctgac gtctaagaaa ccattattat

5641 catgacatta acctataaaa ataggcgtat cacgaggccc ttttgtc

//

**Supplemental File 3**

LOCUS pWus2 4466 bp DNA circular SYN 14-JAN-2025

DEFINITION synthetic circular DNA

ACCESSION .

VERSION .

KEYWORDS .

SOURCE synthetic DNA construct

ORGANISM synthetic DNA construct

REFERENCE 1 (bases 1 to 4466)

AUTHORS .

TITLE Direct Submission

JOURNAL Exported Apr 22, 2025 from SnapGene 8.0.3

https://www.snapgene.com

FEATURES Location/Qualifiers

source 1..4466

/note="/dnas_title=pCMY-ZmWus2 in pUC-GW-Kan"

/organism="synthetic DNA construct"

misc_feature 416..879

/label=CMY 9.11 Promoter

CDS 890..1798

/codon_start=1

/label=ZmWUS2 CDS

/translation="MAANAGGGGAGGGSGSGSVAAPAVCRPSGSRWTPTPEQIRMLKEL

YYGCGIRSPSSEQIQRITAMLRQHGKIEGKNVFYWFQNHKARERQKRRLTSLDVNVPAA

GAADATTSQLGVLSLSSPPPSGAAPPSPTLGFYAAGNGGGSAVLLDTSSDWGSSGAAMA

TETCFLQDYMGVTDTGSSSQWPRFSSSDTIMAAAAARAATTRAPETLPLFPTCGDDGGS

GSSSYLPFWGAASTTAGATSSVAIQQQHQLQEQYSFYSNSNSTQLAGTGNQDVSATAAA

AAALELSLSSWCSPYPAAGSM"

misc_feature 1799..2255

/label=At HSP Term

rep_origin complement(2692..3280)

/direction=LEFT

/label=ori

/note="high-copy-number ColE1/pMB1/pBR322/pUC origin of

replication"

CDS complement(3451..4266)

/codon_start=1

/gene="aph(3')-Ia"

/product="aminoglycoside phosphotransferase"

/label=KanR

/note="confers resistance to kanamycin in bacteria or G418

(Geneticin(R)) in eukaryotes"

/translation="MSHIQRETSCSRPRLNSNMDADLYGYKWARDNVGQSGATIYRLYG

KPDAPELFLKHGKGSVANDVTDEMVRLNWLTEFMPLPTIKHFIRTPDDAWLLTTAIPGK

TAFQVLEEYPDSGENIVDALAVFLRRLHSIPVCNCPFNSDRVFRFAQAQSRMNNGLVDA

SDFDDERNGWPVEQVWKEMHKLLPFSPDSVVTHGDFSLDNLIFDEGKLIGCIDVGRVGI

ADRYQDLAILWNCLGEFSPSLQKRLFQKYGIDNPDMNKLQFHLMLDEFF"

ORIGIN

1 tcgcgcgttt cggtgatgac ggtgaaaacc tctgacacat gcagctcccg gagactgtca

61 cagcttgtct gtaagcggat gccgggagca gacaagcccg tcagggcgcg tcagcgggtg

121 ttggcgggtg tcggggctgg cttaactatg cggcatcaga gcagattgta ctgagagtgc

181 accatatgcg gtgtgaaata ccgcacagat gcgtaaggag aaaataccgc atcaggcgcc

241 attcgccatt caggctgcgc aactgttggg aagggcgatc ggtgcgggcc tcttcgctat

301 tacgccagct ggcgaaaggg ggatgtgctg caaggcgatt aagttgggta acgccagggt

361 tttcccagtc acgacgttgt aaaacgacgg ccagtgaatt gacgcgtatt gggattggca

421 gacatactgt cccacaaatg aagatggaat ctgtaaaaga aaacgcgtga aataatgcgt

481 ctgacaaagg ttaggtcggc tgcctttaat caataccaaa gtggtcccta ccacgatgga

541 aaaactgtgc agtcggtttg gctttttctg acgaacaaat aagattcgtg gccgacaggt

601 gggggtccac catgtgaagg catcttcaga ctccaataat ggagcaatga cgtaagggct

661 tacgaaataa gtaagggtag tttgggaaat gtccactcac ccgtcagtct ataaatactt

721 agcccctccc tcattgttaa gggagcaaaa tctcagagag atagtcctag agagagaaag

781 agagcaagta gcctagaagt agtcaaggcg gcgaagtatt caggcaggtg gccaggaaga

841 agaaaagcca agacgacgaa aacaggtaag agctaagctt tcctgcagga tggcggccaa

901 tgcgggcggc ggtggagcgg gaggaggcag cggcagcggc agcgtggctg cgccggcggt

961 gtgccgcccc agcggctcgc ggtggacgcc gacgccggag cagatcagga tgctgaagga

1021 gctgtactac ggctgcggca tccggtcgcc cagctcggag cagatccagc gcatcaccgc

1081 catgctgcgg cagcacggca agatcgaggg caagaacgtc ttctactggt tccagaacca

1141 caaggcccgc gagcgccaga agcgccgcct caccagcctc gacgtgaacg tgcccgccgc

1201 cggcgcggcc gacgccacca ccagccaact cggcgtcctc tcgctgtcgt cgccgccgcc

1261 ttcaggcgcg gcgcctccct cgcccaccct cggcttctac gccgccggca atggcggcgg

1321 atcggctgtg ctgctggaca cgagttccga ctggggcagc agcggcgctg cgatggccac

1381 cgagacatgc ttcctccagg actacatggg cgtgacggac acgggcagct cgtcgcagtg

1441 gccacgcttc tcgtcgtcgg acacgataat ggcggcggcc gcggcgcggg cggcgacgac

1501 gcgggcgccc gagactctcc ctctcttccc gacctgcggc gacgacggcg gcagcggtag

1561 cagcagctac ttgccgttct ggggtgccgc gtccacaact gccggcgcca cttcttccgt

1621 tgcgatccag cagcaacacc agctgcagga gcagtacagc ttttacagca acagcaacag

1681 cacccagctg gccggcaccg gcaaccaaga cgtatcggca acagcagcag cagccgccgc

1741 cctggagctg agcctcagct catggtgctc cccttaccct gctgcaggga gtatgtgaat

1801 atgaagatga agatgaaata tttggtgtgt caaataaaaa gcttgtgtgc ttaagtttgt

1861 gtttttttct tggcttgttg tgttatgaat ttgtggcttt ttctaatatt aaatgaatgt

1921 aagatctcat tataatgaat aaacaaatgt ttctataatc cattgtgaat gttttgttgg

1981 atctcttctg cagcatataa ctactgtatg tgctatggta tggactatgg aatatgatta

2041 aagataagat gggctcatag agtaaaacga ggcgagggac ctataaacct cccttcatca

2101 tgctatttca tgatctattt tataaaataa agatgtagaa aaaagtaagc gtaataaccg

2161 caaaacaaat gatttaaaac atggcacata atgaggagat taagttcggt ttacgtttat

2221 tttagtacta attgtaacgt gagactacgt atcggatccc aatggcgcgc cgagcttggc

2281 tcgagcatgg tcatagctgt ttcctgtgtg aaattgttat ccgctcacaa ttccacacaa

2341 catacgagcc ggaagcataa agtgtaaagc ctggggtgcc taatgagtga gctaactcac

2401 attaattgcg ttgcgctcac tgcccgcttt ccagtcggga aacctgtcgt gccagctgca

2461 ttaatgaatc ggccaacgcg cggggagagg cggtttgcgt attgggcgct gttccgcttc

2521 ctcgctcact gactcgctgc gctcggtcgt tcggctgcgg cgagcggtat cagctcactc

2581 aaaggcggta atacggttat ccacagaatc aggggataac gcaggaaaga acatgtgagc

2641 aaaaggccag caaaaggcca ggaaccgtaa aaaggccgcg ttgctggcgt ttttccatag

2701 gctccgcccc cctgacgagc atcacaaaaa tcgacgctca agtcagaggt ggcgaaaccc

2761 gacaggacta taaagatacc aggcgtttcc ccctggaagc tccctcgtgc gctctcctgt

2821 tccgaccctg ccgcttaccg gatacctgtc cgcctttctc ccttcgggaa gcgtggcgct

2881 ttctcatagc tcacgctgta ggtatctcag ttcggtgtag gtcgttcgct ccaagctggg

2941 ctgtgtgcac gaaccccccg ttcagcccga ccgctgcgcc ttatccggta actatcgtct

3001 tgagtccaac ccggtaagac acgacttatc gccactggca gcagccactg gtaacaggat

3061 tagcagagcg aggtatgtag gcggtgctac agagttcttg aagtggtggc ctaactacgg

3121 ctacactaga agaacagtat ttggtatctg cgctctgctg aagccagtta ccttcggaaa

3181 aagagttggt agctcttgat ccggcaaaca aaccaccgct ggtagcggtg gtttttttgt

3241 ttgcaagcag cagattacgc gcagaaaaaa aggatctcaa gaagatcctt tgatcttttc

3301 tacggggtct gacgctcagt ggaacgaaaa ctcacgttaa gggattttgg tcatgagatt

3361 atcaaaaagg atcttcacct agatcctttt aaattaaaaa tgaagtttta aatcaatcta

3421 aagtatatat gagtaaactt ggtctgacag ttagaaaaac tcatcgagca tcaaatgaaa

3481 ctgcaattta ttcatatcag gattatcaat accatatttt tgaaaaagcc gtttctgtaa

3541 tgaaggagaa aactcaccga ggcagttcca taggatggca agatcctggt atcggtctgc

3601 gattccgact cgtccaacat caatacaacc tattaatttc ccctcgtcaa aaataaggtt

3661 atcaagtgag aaatcaccat gagtgacgac tgaatccggt gagaatggca aaagtttatg

3721 catttctttc cagacttgtt caacaggcca gccattacgc tcgtcatcaa aatcactcgc

3781 atcaaccaaa ccgttattca ttcgtgattg cgcctgagcg aaacgaaata cgcgatcgct

3841 gttaaaagga caattacaaa caggaatcga atgcaaccgg cgcaggaaca ctgccagcgc

3901 atcaacaata ttttcacctg aatcaggata ttcttctaat acctggaatg ctgttttccc

3961 agggatcgca gtggtgagta accatgcatc atcaggagta cggataaaat gcttgatggt

4021 cggaagaggc ataaattccg tcagccagtt tagtctgacc atctcatctg taacatcatt

4081 ggcaacgcta cctttgccat gtttcagaaa caactctggc gcatcgggct tcccatacaa

4141 tcgatagatt gtcgcacctg attgcccgac attatcgcga gcccatttat acccatataa

4201 atcagcatcc atgttggaat ttaatcgcgg cctagagcaa gacgtttccc gttgaatatg

4261 gctcatactc ttcctttttc aatattattg aagcatttat cagggttatt gtctcatgag

4321 cggatacata tttgaatgta tttagaaaaa taaacaaata ggggttccgc gcacatttcc

4381 ccgaaaagtg ccacctgacg tctaagaaac cattattatc atgacattaa cctataaaaa

4441 taggcgtatc acgaggccct tttgtc

//

**Supplemental File 4**

LOCUS pGRF-GIF 5468 bp DNA circular SYN 14-JAN-2025

DEFINITION synthetic circular DNA

ACCESSION .

VERSION .

KEYWORDS .

SOURCE synthetic DNA construct

ORGANISM synthetic DNA construct

REFERENCE 1 (bases 1 to 5468)

AUTHORS .

TITLE Direct Submission

JOURNAL Exported Apr 22, 2025 from SnapGene 8.0.3

https://www.snapgene.com

FEATURES Location/Qualifiers

source 1..5468

/note="/dnas_title=pCMY-TaGRF4-TaGIF1 in pUC-GW-Kan"

/organism="synthetic DNA construct"

misc_feature 416..879

/label=CMY 9.11 Promoter

CDS 890..2800

/codon_start=1

/label=TaGRF4-TaGIF1 CDS

/translation="MAMPYASLSPAGDRRSSPAATATASLLPFCRSSPFSAGGNGGMGE

EARMDGRWMARPVPFTAAQYEELEHQALIYKYLVAGVSVPPDLVLPIRRGIESLAARFY

HNPLAIGYGSYLGKKVDPEPGRCRRTDGKKWRCAKEAASDSKYCERHMHRGRNRSRKPV

ETQLVSHSQPPAASVVPPLATGFHNHSLYPAIGGTNGGGGGGNNGMSMPGTFSSALGPP

QQHMGNNAASPYAALGGAGTCKDFRYTAYGIRSLADEQSQLMTEAMNTSVENPWRLPPS

SQTTTFPLSSYSPQLGATSDLGQNNSSNNNSGVKAEGQQQQQPLSFPGCGDFGSGDSAK

QENQTLRPFFDEWPKTRDSWSDLTDDNSNVASFSATQLSISIPMTSSDFSAASSQSPNG

MLFAGEMYAAAAMQQQHLMQMNQSMMGGYASSTTATTDLIQQYLDENKQLILAILDNQN

NGKVEECARNQAKLQQNLMYLAAIADSQPPQTASLSQYPSNLMMQSGPRYMQQQSAQMM

SPQSLMAARSSMMYAQQAMSPLQQQQQQQQHQAAAHGQLGMSSGATTGFNLLHGEASMG

GGGGATGNSMMNASVFSDYGRGGSGAKEGSTSLSADARGANSGAHSGDGEYLKGTEEEG

S"

misc_feature 2801..3257

/label=At HSP Term

rep_origin complement(3694..4282)

/direction=LEFT

/label=ori

/note="high-copy-number ColE1/pMB1/pBR322/pUC origin of

replication"

CDS complement(4453..5268)

/codon_start=1

/gene="aph(3')-Ia"

/product="aminoglycoside phosphotransferase"

/label=KanR

/note="confers resistance to kanamycin in bacteria or G418

(Geneticin(R)) in eukaryotes"

/translation="MSHIQRETSCSRPRLNSNMDADLYGYKWARDNVGQSGATIYRLYG

KPDAPELFLKHGKGSVANDVTDEMVRLNWLTEFMPLPTIKHFIRTPDDAWLLTTAIPGK

TAFQVLEEYPDSGENIVDALAVFLRRLHSIPVCNCPFNSDRVFRFAQAQSRMNNGLVDA

SDFDDERNGWPVEQVWKEMHKLLPFSPDSVVTHGDFSLDNLIFDEGKLIGCIDVGRVGI

ADRYQDLAILWNCLGEFSPSLQKRLFQKYGIDNPDMNKLQFHLMLDEFF"

ORIGIN

1 tcgcgcgttt cggtgatgac ggtgaaaacc tctgacacat gcagctcccg gagactgtca

61 cagcttgtct gtaagcggat gccgggagca gacaagcccg tcagggcgcg tcagcgggtg

121 ttggcgggtg tcggggctgg cttaactatg cggcatcaga gcagattgta ctgagagtgc

181 accatatgcg gtgtgaaata ccgcacagat gcgtaaggag aaaataccgc atcaggcgcc

241 attcgccatt caggctgcgc aactgttggg aagggcgatc ggtgcgggcc tcttcgctat

301 tacgccagct ggcgaaaggg ggatgtgctg caaggcgatt aagttgggta acgccagggt

361 tttcccagtc acgacgttgt aaaacgacgg ccagtgaatt gacgcgtatt gggattggca

421 gacatactgt cccacaaatg aagatggaat ctgtaaaaga aaacgcgtga aataatgcgt

481 ctgacaaagg ttaggtcggc tgcctttaat caataccaaa gtggtcccta ccacgatgga

541 aaaactgtgc agtcggtttg gctttttctg acgaacaaat aagattcgtg gccgacaggt

601 gggggtccac catgtgaagg catcttcaga ctccaataat ggagcaatga cgtaagggct

661 tacgaaataa gtaagggtag tttgggaaat gtccactcac ccgtcagtct ataaatactt

721 agcccctccc tcattgttaa gggagcaaaa tctcagagag atagtcctag agagagaaag

781 agagcaagta gcctagaagt agtcaaggcg gcgaagtatt caggcaggtg gccaggaaga

841 agaaaagcca agacgacgaa aacaggtaag agctaagctt tcctgcagga tggcaatgcc

901 ctacgcctct ctttcgcccg ctggcgatcg taggtcgtca cccgcagcga ctgcgactgc

961 gtccctcctc cctttctgcc gttcatcgcc cttctcggca ggcggcaatg ggggtatggg

1021 agaagaagca aggatggacg ggagatggat ggcaagacct gtgccgttta ccgctgcgca

1081 atatgaagaa ttggaacatc aggcgctcat ttataaatat cttgttgccg gggtaagtgt

1141 ccccccggac cttgtgctgc ccatccgtag gggtatagag tcacttgcgg ctcgttttta

1201 tcataaccct ttggctattg gctatggatc gtacttgggt aagaaggtgg acccggagcc

1261 gggcaggtgc cgtagaactg atggtaagaa gtggagatgt gcgaaggaag ccgcttcgga

1321 ctccaaatat tgtgaacgtc atatgcatcg tgggaggaat agatctagga aaccagtaga

1381 gactcaactg gtatcccact cccagccccc tgcggcgagc gtggtgccgc cgttggctac

1441 cggcttccat aaccattctt tgtatccagc gattggaggc actaatggtg gagggggcgg

1501 cggtaataac gggatgtcaa tgcctgggac attcagttca gctcttggcc ccccacagca

1561 acatatggga aacaatgccg ctagcccgta cgcagccctg ggtggggctg gtacctgcaa

1621 ggatttcaga tatacggcgt acggcataag atccctggca gatgagcagt cccagcttat

1681 gactgaggcc atgaacacca gtgtcgagaa cccatggcgt ttgcctcctt caagccaaac

1741 gacaaccttt ccgttgagct catattcacc ccagctcgga gcaacgtcgg acctcggtca

1801 aaacaactcc agtaataata atagtggtgt taaagcggaa ggacagcagc agcagcaacc

1861 gctttcattc cctggctgcg gtgactttgg atccggggat tcggcgaagc aagaaaatca

1921 gacgctcaga cccttctttg acgagtggcc aaagacgaga gactcatggt cggatcttac

1981 agacgataac tctaatgtcg cgagtttctc cgccacacag ctcagtatat cgattcccat

2041 gacttcatcg gattttagcg ccgcaagttc tcaatcgccc aacggaatgc ttttcgctgg

2101 cgaaatgtat gctgccgccg cgatgcagca gcagcatctc atgcagatga accagtcaat

2161 gatgggtgga tacgctagca gtacaactgc aaccacggat cttattcaac agtatttgga

2221 tgaaaataag caacttatat tggctattct ggataaccag aataatggta aggtcgaaga

2281 gtgcgctcgt aatcaagcta agctccagca gaatctcatg tatctcgcag ctatagctga

2341 ttctcagcct cctcagactg cttccctctc tcagtatccc agcaacttga tgatgcaatc

2401 tggcccaagg tatatgcagc aacagtccgc acaaatgatg agcccccaat cgttgatggc

2461 ggcgcgttcg tccatgatgt atgcgcaaca ggcaatgagt cctttgcagc aacaacagca

2521 acaacaacag catcaggcgg ccgcacacgg acagctcgga atgtcttcgg gtgctaccac

2581 cggattcaac ttgcttcatg gtgaggcgtc tatggggggc ggtggggggg cgacgggcaa

2641 ttctatgatg aacgcttcgg ttttctcgga ctacgggcgt ggtggaagtg gcgctaaaga

2701 aggttccacc tccctgagtg cggacgcaag gggagccaat tccggtgcgc attctggcga

2761 tggggaatac cttaaaggta ctgaggagga aggaagctga atatgaagat gaagatgaaa

2821 tatttggtgt gtcaaataaa aagcttgtgt gcttaagttt gtgttttttt cttggcttgt

2881 tgtgttatga atttgtggct ttttctaata ttaaatgaat gtaagatctc attataatga

2941 ataaacaaat gtttctataa tccattgtga atgttttgtt ggatctcttc tgcagcatat

3001 aactactgta tgtgctatgg tatggactat ggaatatgat taaagataag atgggctcat

3061 agagtaaaac gaggcgaggg acctataaac ctcccttcat catgctattt catgatctat

3121 tttataaaat aaagatgtag aaaaaagtaa gcgtaataac cgcaaaacaa atgatttaaa

3181 acatggcaca taatgaggag attaagttcg gtttacgttt attttagtac taattgtaac

3241 gtgagactac gtatcggatc ccaatggcgc gccgagcttg gctcgagcat ggtcatagct

3301 gtttcctgtg tgaaattgtt atccgctcac aattccacac aacatacgag ccggaagcat

3361 aaagtgtaaa gcctggggtg cctaatgagt gagctaactc acattaattg cgttgcgctc

3421 actgcccgct ttccagtcgg gaaacctgtc gtgccagctg cattaatgaa tcggccaacg

3481 cgcggggaga ggcggtttgc gtattgggcg ctgttccgct tcctcgctca ctgactcgct

3541 gcgctcggtc gttcggctgc ggcgagcggt atcagctcac tcaaaggcgg taatacggtt

3601 atccacagaa tcaggggata acgcaggaaa gaacatgtga gcaaaaggcc agcaaaaggc

3661 caggaaccgt aaaaaggccg cgttgctggc gtttttccat aggctccgcc cccctgacga

3721 gcatcacaaa aatcgacgct caagtcagag gtggcgaaac ccgacaggac tataaagata

3781 ccaggcgttt ccccctggaa gctccctcgt gcgctctcct gttccgaccc tgccgcttac

3841 cggatacctg tccgcctttc tcccttcggg aagcgtggcg ctttctcata gctcacgctg

3901 taggtatctc agttcggtgt aggtcgttcg ctccaagctg ggctgtgtgc acgaaccccc

3961 cgttcagccc gaccgctgcg ccttatccgg taactatcgt cttgagtcca acccggtaag

4021 acacgactta tcgccactgg cagcagccac tggtaacagg attagcagag cgaggtatgt

4081 aggcggtgct acagagttct tgaagtggtg gcctaactac ggctacacta gaagaacagt

4141 atttggtatc tgcgctctgc tgaagccagt taccttcgga aaaagagttg gtagctcttg

4201 atccggcaaa caaaccaccg ctggtagcgg tggttttttt gtttgcaagc agcagattac

4261 gcgcagaaaa aaaggatctc aagaagatcc tttgatcttt tctacggggt ctgacgctca

4321 gtggaacgaa aactcacgtt aagggatttt ggtcatgaga ttatcaaaaa ggatcttcac

4381 ctagatcctt ttaaattaaa aatgaagttt taaatcaatc taaagtatat atgagtaaac

4441 ttggtctgac agttagaaaa actcatcgag catcaaatga aactgcaatt tattcatatc

4501 aggattatca ataccatatt tttgaaaaag ccgtttctgt aatgaaggag aaaactcacc

4561 gaggcagttc cataggatgg caagatcctg gtatcggtct gcgattccga ctcgtccaac

4621 atcaatacaa cctattaatt tcccctcgtc aaaaataagg ttatcaagtg agaaatcacc

4681 atgagtgacg actgaatccg gtgagaatgg caaaagttta tgcatttctt tccagacttg

4741 ttcaacaggc cagccattac gctcgtcatc aaaatcactc gcatcaacca aaccgttatt

4801 cattcgtgat tgcgcctgag cgaaacgaaa tacgcgatcg ctgttaaaag gacaattaca

4861 aacaggaatc gaatgcaacc ggcgcaggaa cactgccagc gcatcaacaa tattttcacc

4921 tgaatcagga tattcttcta atacctggaa tgctgttttc ccagggatcg cagtggtgag

4981 taaccatgca tcatcaggag tacggataaa atgcttgatg gtcggaagag gcataaattc

5041 cgtcagccag tttagtctga ccatctcatc tgtaacatca ttggcaacgc tacctttgcc

5101 atgtttcaga aacaactctg gcgcatcggg cttcccatac aatcgataga ttgtcgcacc

5161 tgattgcccg acattatcgc gagcccattt atacccatat aaatcagcat ccatgttgga

5221 atttaatcgc ggcctagagc aagacgtttc ccgttgaata tggctcatac tcttcctttt

5281 tcaatattat tgaagcattt atcagggtta ttgtctcatg agcggataca tatttgaatg

5341 tatttagaaa aataaacaaa taggggttcc gcgcacattt ccccgaaaag tgccacctga

5401 cgtctaagaa accattatta tcatgacatt aacctataaa aataggcgta tcacgaggcc

5461 cttttgtc

//

**Supplemental File 5**

LOCUS pHYG 4583 bp DNA circular SYN 14-JAN-2025

DEFINITION synthetic circular DNA

ACCESSION .

VERSION .

KEYWORDS .

SOURCE synthetic DNA construct

ORGANISM synthetic DNA construct

REFERENCE 1 (bases 1 to 4583)

AUTHORS .

TITLE Direct Submission

JOURNAL Exported Apr 22, 2025 from SnapGene 8.0.3

https://www.snapgene.com

FEATURES Location/Qualifiers

source 1..4583

/note="/dnas_title=CMY-HYG in pUC-GW-Kan"

/organism="synthetic DNA construct"

misc_feature 416..879

/label=CMY 9.11 Promoter

CDS 890..1915

/codon_start=1

/label=HYG CDS

/translation="MKKPELTATSVEKFLIEKFDSVSDLMQLSEGEESRAFSFDVGGRG

YVLRVNSCADGFYKDRYVYRHFASAALPIPEVLDIGEFSESLTYCISRRAQGVTLQDLP

ETELPAVLQPVAEAMDAIAAADLSQTSGFGPFGPQGIGQYTTWRDFICAIADPHVYHWQ

TVMDDTVSASVAQALDELMLWAEDCPEVRHLVHADFGSNNVLTDNGRITAVIDWSEAMF

GDSQYEVANIFFWRPWLACMEQQTRYFERRHPELAGSPRLRAYMLRIGLDQLYQSLVDG

NFDDAAWAQGRCDAIVRSGAGTVGRTQIARRSAAVWTDGCVEVLADSGNRRPSTRPRAK

K"

misc_feature 1916..2372

/label=At HSP Term

rep_origin complement(2809..3397)

/direction=LEFT

/label=ori

/note="high-copy-number ColE1/pMB1/pBR322/pUC origin of

replication"

CDS complement(3568..4383)

/codon_start=1

/gene="aph(3')-Ia"

/product="aminoglycoside phosphotransferase"

/label=KanR

/note="confers resistance to kanamycin in bacteria or G418

(Geneticin(R)) in eukaryotes"

/translation="MSHIQRETSCSRPRLNSNMDADLYGYKWARDNVGQSGATIYRLYG

KPDAPELFLKHGKGSVANDVTDEMVRLNWLTEFMPLPTIKHFIRTPDDAWLLTTAIPGK

TAFQVLEEYPDSGENIVDALAVFLRRLHSIPVCNCPFNSDRVFRFAQAQSRMNNGLVDA

SDFDDERNGWPVEQVWKEMHKLLPFSPDSVVTHGDFSLDNLIFDEGKLIGCIDVGRVGI

ADRYQDLAILWNCLGEFSPSLQKRLFQKYGIDNPDMNKLQFHLMLDEFF"

ORIGIN

1 tcgcgcgttt cggtgatgac ggtgaaaacc tctgacacat gcagctcccg gagactgtca

61 cagcttgtct gtaagcggat gccgggagca gacaagcccg tcagggcgcg tcagcgggtg

121 ttggcgggtg tcggggctgg cttaactatg cggcatcaga gcagattgta ctgagagtgc

181 accatatgcg gtgtgaaata ccgcacagat gcgtaaggag aaaataccgc atcaggcgcc

241 attcgccatt caggctgcgc aactgttggg aagggcgatc ggtgcgggcc tcttcgctat

301 tacgccagct ggcgaaaggg ggatgtgctg caaggcgatt aagttgggta acgccagggt

361 tttcccagtc acgacgttgt aaaacgacgg ccagtgaatt gacgcgtatt gggattggca

421 gacatactgt cccacaaatg aagatggaat ctgtaaaaga aaacgcgtga aataatgcgt

481 ctgacaaagg ttaggtcggc tgcctttaat caataccaaa gtggtcccta ccacgatgga

541 aaaactgtgc agtcggtttg gctttttctg acgaacaaat aagattcgtg gccgacaggt

601 gggggtccac catgtgaagg catcttcaga ctccaataat ggagcaatga cgtaagggct

661 tacgaaataa gtaagggtag tttgggaaat gtccactcac ccgtcagtct ataaatactt

721 agcccctccc tcattgttaa gggagcaaaa tctcagagag atagtcctag agagagaaag

781 agagcaagta gcctagaagt agtcaaggcg gcgaagtatt caggcaggtg gccaggaaga

841 agaaaagcca agacgacgaa aacaggtaag agctaagctt tcctgcagga tgaagaagcc

901 tgagttgacc gccacttctg tcgagaaatt tctgatcgag aagtttgatt cggtgagcga

961 tctgatgcag ctttcggagg gcgaggagtc acgcgcgttc tccttcgatg ttggcgggcg

1021 gggatatgtg cttcgggtta actcttgtgc ggacggtttc tacaaagatc gctacgtcta

1081 tcgccacttt gccagcgcag ccttgcctat ccccgaagtt ttggacattg gtgaattctc

1141 cgaatcactg acatattgta tctcacgccg ggcgcagggt gttacgctcc aggacctccc

1201 tgaaactgaa ttgcctgcgg tgctccagcc ggtggctgag gcgatggatg cgatagctgc

1261 tgctgacctc tcccaaacgt ccggatttgg tccattcggg ccacagggca tcggccagta

1321 cactacatgg cgcgatttta tctgcgcaat tgccgacccc catgtgtacc actggcaaac

1381 ggtgatggat gacacagtta gcgcatccgt cgcgcaggct ctcgacgaac ttatgctctg

1441 ggccgaagat tgtcccgagg ttcggcactt ggttcatgcg gattttgggt ccaacaacgt

1501 gctcaccgac aacggcagga ttacggctgt tatagactgg tcagaggcta tgtttggaga

1561 ttcgcaatat gaggtcgcta atatcttctt ttggcgcccc tggcttgcct gtatggaaca

1621 gcaaacgaga tactttgaaa gacgccaccc agagctggcg ggatcccctc gcttgcgggc

1681 ttacatgttg agaatcggtc ttgaccagct gtaccagagc ctcgtggacg gtaacttcga

1741 tgatgccgct tgggcgcagg gaagatgcga tgcaattgtt aggtccggag ctggaaccgt

1801 gggccgcacg cagattgcaa gaagatctgc tgccgtttgg acagatggct gtgttgaagt

1861 gcttgcagac tccgggaacc ggaggccaag cacacggccg agggctaaga aatgaatatg

1921 aagatgaaga tgaaatattt ggtgtgtcaa ataaaaagct tgtgtgctta agtttgtgtt

1981 tttttcttgg cttgttgtgt tatgaatttg tggctttttc taatattaaa tgaatgtaag

2041 atctcattat aatgaataaa caaatgtttc tataatccat tgtgaatgtt ttgttggatc

2101 tcttctgcag catataacta ctgtatgtgc tatggtatgg actatggaat atgattaaag

2161 ataagatggg ctcatagagt aaaacgaggc gagggaccta taaacctccc ttcatcatgc

2221 tatttcatga tctattttat aaaataaaga tgtagaaaaa agtaagcgta ataaccgcaa

2281 aacaaatgat ttaaaacatg gcacataatg aggagattaa gttcggttta cgtttatttt

2341 agtactaatt gtaacgtgag actacgtatc ggatcccaat ggcgcgccga gcttggctcg

2401 agcatggtca tagctgtttc ctgtgtgaaa ttgttatccg ctcacaattc cacacaacat

2461 acgagccgga agcataaagt gtaaagcctg gggtgcctaa tgagtgagct aactcacatt

2521 aattgcgttg cgctcactgc ccgctttcca gtcgggaaac ctgtcgtgcc agctgcatta

2581 atgaatcggc caacgcgcgg ggagaggcgg tttgcgtatt gggcgctgtt ccgcttcctc

2641 gctcactgac tcgctgcgct cggtcgttcg gctgcggcga gcggtatcag ctcactcaaa

2701 ggcggtaata cggttatcca cagaatcagg ggataacgca ggaaagaaca tgtgagcaaa

2761 aggccagcaa aaggccagga accgtaaaaa ggccgcgttg ctggcgtttt tccataggct

2821 ccgcccccct gacgagcatc acaaaaatcg acgctcaagt cagaggtggc gaaacccgac

2881 aggactataa agataccagg cgtttccccc tggaagctcc ctcgtgcgct ctcctgttcc

2941 gaccctgccg cttaccggat acctgtccgc ctttctccct tcgggaagcg tggcgctttc

3001 tcatagctca cgctgtaggt atctcagttc ggtgtaggtc gttcgctcca agctgggctg

3061 tgtgcacgaa ccccccgttc agcccgaccg ctgcgcctta tccggtaact atcgtcttga

3121 gtccaacccg gtaagacacg acttatcgcc actggcagca gccactggta acaggattag

3181 cagagcgagg tatgtaggcg gtgctacaga gttcttgaag tggtggccta actacggcta

3241 cactagaaga acagtatttg gtatctgcgc tctgctgaag ccagttacct tcggaaaaag

3301 agttggtagc tcttgatccg gcaaacaaac caccgctggt agcggtggtt tttttgtttg

3361 caagcagcag attacgcgca gaaaaaaagg atctcaagaa gatcctttga tcttttctac

3421 ggggtctgac gctcagtgga acgaaaactc acgttaaggg attttggtca tgagattatc

3481 aaaaaggatc ttcacctaga tccttttaaa ttaaaaatga agttttaaat caatctaaag

3541 tatatatgag taaacttggt ctgacagtta gaaaaactca tcgagcatca aatgaaactg

3601 caatttattc atatcaggat tatcaatacc atatttttga aaaagccgtt tctgtaatga

3661 aggagaaaac tcaccgaggc agttccatag gatggcaaga tcctggtatc ggtctgcgat

3721 tccgactcgt ccaacatcaa tacaacctat taatttcccc tcgtcaaaaa taaggttatc

3781 aagtgagaaa tcaccatgag tgacgactga atccggtgag aatggcaaaa gtttatgcat

3841 ttctttccag acttgttcaa caggccagcc attacgctcg tcatcaaaat cactcgcatc

3901 aaccaaaccg ttattcattc gtgattgcgc ctgagcgaaa cgaaatacgc gatcgctgtt

3961 aaaaggacaa ttacaaacag gaatcgaatg caaccggcgc aggaacactg ccagcgcatc

4021 aacaatattt tcacctgaat caggatattc ttctaatacc tggaatgctg ttttcccagg

4081 gatcgcagtg gtgagtaacc atgcatcatc aggagtacgg ataaaatgct tgatggtcgg

4141 aagaggcata aattccgtca gccagtttag tctgaccatc tcatctgtaa catcattggc

4201 aacgctacct ttgccatgtt tcagaaacaa ctctggcgca tcgggcttcc catacaatcg

4261 atagattgtc gcacctgatt gcccgacatt atcgcgagcc catttatacc catataaatc

4321 agcatccatg ttggaattta atcgcggcct agagcaagac gtttcccgtt gaatatggct

4381 catactcttc ctttttcaat attattgaag catttatcag ggttattgtc tcatgagcgg

4441 atacatattt gaatgtattt agaaaaataa acaaataggg gttccgcgca catttccccg

4501 aaaagtgcca cctgacgtct aagaaaccat tattatcatg acattaacct ataaaaatag

4561 gcgtatcacg aggccctttt gtc

//
